# Characterization of Visceral and Subcutaneous Adipose Tissue Transcriptome and Biological Pathways in Pregnant and Non-Pregnant Women: Evidence for Pregnancy-Related Regional-Specific Differences in Adipose Tissue

**DOI:** 10.1371/journal.pone.0143779

**Published:** 2015-12-04

**Authors:** Shali Mazaki-Tovi, Edi Vaisbuch, Adi L. Tarca, Juan Pedro Kusanovic, Nandor Gabor Than, Tinnakorn Chaiworapongsa, Zhong Dong, Sonia S. Hassan, Roberto Romero

**Affiliations:** 1 Department of Obstetrics and Gynecology, Sheba Medical Center, Tel Hashomer, Israel; 2 Tel Aviv University, Tel Aviv, Israel; 3 Department of Obstetrics and Gynecology, Kaplan Medical Center, Rehovot, Israel; 4 Perinatology Research Branch, Eunice Kennedy Shriver National Institute of Child Health and Human Development, National Institutes of Health, Department of Health and Human Services, Bethesda, Maryland, and Detroit, Michigan, United States of America; 5 Department of Computer Science, Wayne State University, Detroit, Michigan, United States of America; 6 Center for Molecular Medicine and Genetics, Wayne State University School of Medicine, Detroit, Michigan, United States of America; 7 Department of Obstetrics and Gynecology, School of Medicine, Pontificia Universidad Católica de Chile, Santiago, Chile; 8 Center for Research and Innovation in Maternal-Fetal Medicine (CIMAF), Department of Obstetrics and Gynecology, Sótero del Río Hospital, Santiago, Chile; 9 Institute of Enzymology, Research Centre for Natural Sciences, Hungarian Academy of Sciences, Budapest, Hungary; 10 First Department of Pathology and Experimental Cancer Research, Semmelweis University, Budapest, Hungary; 11 Department of Obstetrics and Gynecology, Wayne State University School of Medicine, Detroit, Michigan, United States of America; Virgen Macarena University Hospital, School of Medicine, University of Seville, SPAIN

## Abstract

**Objective:**

The purpose of this study was to compare the transcriptome of visceral and subcutaneous adipose tissues between pregnant and non-pregnant women.

**Study Design:**

The transcriptome of paired visceral and abdominal subcutaneous adipose tissues from pregnant women at term and matched non-pregnant women (n = 11) was profiled with the Affymetrix Human Exon 1.0 ST array. Differential expression of selected genes was validated with the use of quantitative reverse transcription–polymerase chain reaction.

**Results:**

Six hundred forty-four transcripts from 633 known genes were differentially expressed (false discovery rate (FDR) <0.1; fold-change >1.5), while 42 exons from 36 genes showed differential usage (difference in FIRMA scores >2 and FDR<0.1) between the visceral and subcutaneous fat of pregnant women. Fifty-six known genes were differentially expressed between pregnant and non-pregnant subcutaneous fat and three genes in the visceral fat. Enriched biological processes in the subcutaneous adipose tissue of pregnant women were mostly related to inflammation.

**Conclusion:**

The transcriptome of visceral and subcutaneous fat depots reveals pregnancy-related gene expression and splicing differences in both visceral and subcutaneous adipose tissue. Furthermore, for the first time, alternative splicing in adipose tissue has been associated with regional differences and human parturition.

## Introduction

Physiological adaptations of normal pregnancy include insulin resistance,[1–3] hyperlipidemia,[4,5] and, most notably, increased fat depot.[6,7] Teleologically, these profound metabolic changes are aimed to ensure adequate nutrient supply for the rapidly growing fetus and placenta. The ephemeral nature of the pregnancy-induced metabolic alterations, as well as empirical findings,[8–11] led to the conventional view that these physiologic adaptations stem solely from the “diabetogenic” effect of the placental hormones. This concept has been challenged by a large body of evidence suggesting that adipose tissue may play a regulatory role in both normal and abnormal gestations.

During the last decade, adipose tissue has emerged as a powerful endocrine organ [12–17] that exerts autocrine, paracrine, and endocrine effects by production and secretion of highly active peptides and proteins collectively termed adipokines.[15,18] The realization that fat is an important endocrine organ that is crucial for whole-body insulin sensitivity and energy homeostasis has rekindled the scientific interest in adipose tissue in both non-pregnant and pregnant individuals. Indeed, adipokines have been implicated in physiological adaptations of normal gestation,[12,19–34] as well as in the pathophysiology of preeclampsia,[35–54] gestational diabetes mellitus,[55–72] preterm birth,[73–75] delivery of large-for-gestational-age (LGA) newborns,[76] small-for-gestational-age (SGA)[77–84] neonates, pyelonephritis,[85–87] and intrauterine infection and inflammation.[88–91] Of note is the well established association between obesity and theses complications of pregnancy.[30–33,70–72,92–117]

An emerging concept is that fat accrual in different depots is associated with different metabolic consequences.[118] Specifically, accumulation of visceral (intra-abdominal) fat is associated with a much higher risk of diabetes, dyslipidemia, accelerated atherosclerosis, and metabolic syndrome than subcutaneous fat accretion.[119–122] Although the specific mechanism(s) by which an intra-abdominal fat depot exerts its detrimental effects has not been fully elucidated, it is clear that visceral and subcutaneous adipose tissues display distinct structural and functional properties, which include: 1) the size of adipocytes is smaller in visceral than in subcutaneous adipose tissue;[123] 2) visceral fat cells have an increased β-adrenoceptor-mediated lipolysis;[124] 3) visceral adipocytes display greater responsiveness to both adrenergic receptor- and postreceptor-acting agents compared with subcutaneous adipocytes[123]; 4) gene expression of β1- and β3-adrenoceptors is higher and β2-adrenoceptor lower in the visceral cells;[124] and 5) mRNA expression[125] and secretion of important adipokines such as leptin,[125,126] adiponectin,[126,127] and retinol-binding protein-4 (RBP4),[126] is lower in visceral compared to subcutaneous adipocytes.

The mechanisms responsible for adipose tissue depot-specific structural and functional differences are unknown. Regional variations in specific genes coding for important functional proteins[124,126–148] have led several investigators to employ high throughput techniques to identify adipose tissue depot-specific gene differences in non-pregnant individuals.[128,131,149–151] However, to date, no studies have been published on the differences in the transcriptome of visceral and subcutaneous adipose tissues between pregnant and non-pregnant women. Furthermore, to our knowledge, alternative splice variants whose expression differs between visceral and subcutaneous have not been reported in either pregnant or non-pregnant individuals.

This study was undertaken to characterize the transcriptome of visceral and subcutaneous adipose tissues during human pregnancy to gain further insight into the molecular changes that are associated with normal gestation. The aims of this study were: 1) to determine the differences between visceral and subcutaneous gene expression in non-pregnant women; 2) to characterize, for the first time, regional variations in the transcriptome of adipose tissue during normal pregnancy; 3) to determine the differences in visceral and subcutaneous gene expression between pregnant and non-pregnant women; and 4) to identify depot-specific and pregnancy-related alternative splicing alterations in adipose tissue.

## Materials and Methods

### Study groups

A prospective study was performed in which visceral and subcutaneous adipose tissue samples were obtained from patients in the following groups: 1) pregnant women undergoing elective cesarean section at term (n = 25); and 2) non-pregnant women undergoing elective laparotomy for conservative myomectomy (n = 11). Patients were matched according to age, parity and body mass index (BMI) at sampling for the comparison between pregnant and non-pregnant women.

The inclusion criteria for both groups were: 1) absence of medical complications; 2) no antibiotic administration prior to the sample collection; and 3) normal post-operative course. The inclusion criteria for pregnant women also included: 1) absence of obstetric complications of pregnancy; 2) normal pregnancy outcome, including an infant who was of appropriate weight for gestational age (AGA) without congenital anomalies and had Apgar scores >7 at 1 and 5 minutes; 3) absence of meconium staining of the amniotic fluid; and 4) absence of histologic chorioamnionitis.

Eligible patients were enrolled at Hutzel Women’s Hospital (Detroit, MI, USA). All women provided written informed consent prior to the collection of adipose tissue samples. The collection and utilization of the samples for research purposes were approved by the Institutional Review Board of the *Eunice Kennedy Shriver* National Institute of Child Health and Human Development (NICHD), National Institutes of Health (NIH), U.S. Department of Health and Human Services (DHHS), Bethesda, MD, and Detroit, MI, USA), and the Human Investigation Committee of Wayne State University (Detroit, MI, USA).

### Clinical definitions

Patients not in labor underwent a cesarean section secondary to a fetus in non-cephalic presentation, a previous uterine surgery or classical cesarean section, or an elective cesarean section with no more than one previous cesarean section. Only women who delivered an AGA newborn were included. Acute histologic chorioamnionitis was diagnosed using previously described criteria.[152,153] An AGA neonate was defined as having a birth weight between the 10^th^ and 90^th^ percentiles for the gestational age at birth.[154] Term gestation was defined as gestational age >37 completed weeks. BMI was calculated according to the formula: weight (kg)/height (m^2^). Normal weight was defined as a BMI of 18.5–24.9 kg/m^2^, overweight as a BMI 25–29.9 kg/m^2^, and obesity as a BMI >30 kg/m^2^, according to the definitions of the World Health Organization.[155]

### Sample collection

Paired visceral and subcutaneous adipose tissue samples were obtained after an eight-hour fast. Subcutaneous adipose tissue samples were collected at the site of a transverse lower abdominal incision, in the middle of the Pfannenstiel incision, from the deeper strata of subcutaneous fat. Visceral samples were obtained from the most distal portion of the greater omentum.[127,156–159] Visceral and subcutaneous adipose tissues were collected using Metzenbaum scissors and measured approximately 1.0 cm^3^. Tissues were snap-frozen in liquid nitrogen, and were kept at –80°C until use.

### RNA isolation

Total RNA was isolated from snap-frozen adipose tissue samples using TRI Reagent^®^ (Ambion^®^, Life Technologies Corporation, Austin, TX, USA) combined with the Qiagen RNeasy Lipid Tissue Kit protocol (Qiagen, Valencia, CA, USA), according to the manufacturers’ recommendations. The RNA concentrations and the A260 nm/A280 nm ratios were assessed using a NanoDrop^®^ 1000 Spectrophotometer (Thermo Scientific, Wilmington, DE, USA). RNA integrity numbers were determined using the Agilent Bioanalyzer 2100 (Agilent Technologies, Wilmington, DE, USA).

### Microarray analysis and quantitative real-time reverse-transcription polymerase chain reaction (qRT-PCR)

The Affymetrix GeneChip Human Exon 1.0 ST array (Affymetrix, Santa Clara, CA, USA) platform was used to measure the expression levels in each unpooled specimen, according to the manufacturer's instructions. The array contains approximately 5.4 million 5-μm features (probes) grouped into 1.4 million probesets interrogating more than one million exon clusters.[160–162] To verify the results from microarray, 53 genes were selected for qRT-PCR assays from the original sample set (n = 11). A detailed description of the method and analysis is available as supplementary material (S1 File. Supplementary methods).

### Statistical analyses

The raw gene expression data were preprocessed using Robust Multi-array Average (RMA).[163] A paired moderated *t* test[164] was used to test for differential expression with a false discovery rate (FDR)[165] threshold of 0.1 in conjunction with a threshold of 1.5 on the fold-change to assign gene significance.[166] Differential exon usage was tested using the FIRMA (Finding Isoforms Using Robust Multichip Analysis) method [167] adapted for multiple samples as described in the Supplementary methods (S1 File). Gene Ontology analysis was conducted with algorithms that were described previously.[168] Pathway analysis was performed on the Kyoto Encyclopedia of Genes and Genomes (KEGG)[169] pathway database with an overrepresentation analysis[170] Assessment of differential expression between experimental regions from qRT-PCR data was performed with a paired *t* test on –ΔCt values. The Student *t*, Mann-Whitney *U*, and X^2^ tests were used to identify significant differences in patient demographics between women in the microarray and qRT-PCR groups. SPSS software (version 14.0; SPSS Inc, Chicago, IL) was used for statistical analysis of the demographic data. A probability value of < 0.05 was considered statistically significant.

## Results

### Demographics

Table 1 displays the demographic characteristics of patients who were included in the microarray and qRT-PCR analyses.

**Table 1:** Demographic and clinical characteristics.

	Pregnant N = 25	Non-Pregnant N = 11	p
**Maternal age (years)**	34 (33–42)	32 (28–40)	0.6
**BMI at Sampling (kg/m2)**	30.4 (27.5–38.8)	31.1 (26.2–39.1)	0.9
**Gravidity**	4 (3–5)	4 (3–7)	0.7
**Parity**	3 (2–4)	3 (2–4)	0.6
**Ethnic Origin (%)**			0.5
** African American**	90.9	81.8	
** Caucasian**	9.1	18.2	
**Systolic Blood Pressure (mmHg)**	117 (114–120)	114 (111–117)	0.6
**Diastolic Blood Pressure (mmHg)**	74 (69–79)	66 (63–81)	0.4
**Fasting Glucose (mg/dl)**	87 (80–89)	88 (73–90)	0.3
**Gestational Age at Delivery (weeks)**	39.1 (38–39.3)	NA	NA
**Birth Weight (grams)**	3335 (2980–3555)	NA	NA

Data are presented as median and interquartile range (IQR). BMI—Body Mass Index.

### Results of the microarray analysis

#### Pregnant women: visceral versus subcutaneous

**Fig 1:**
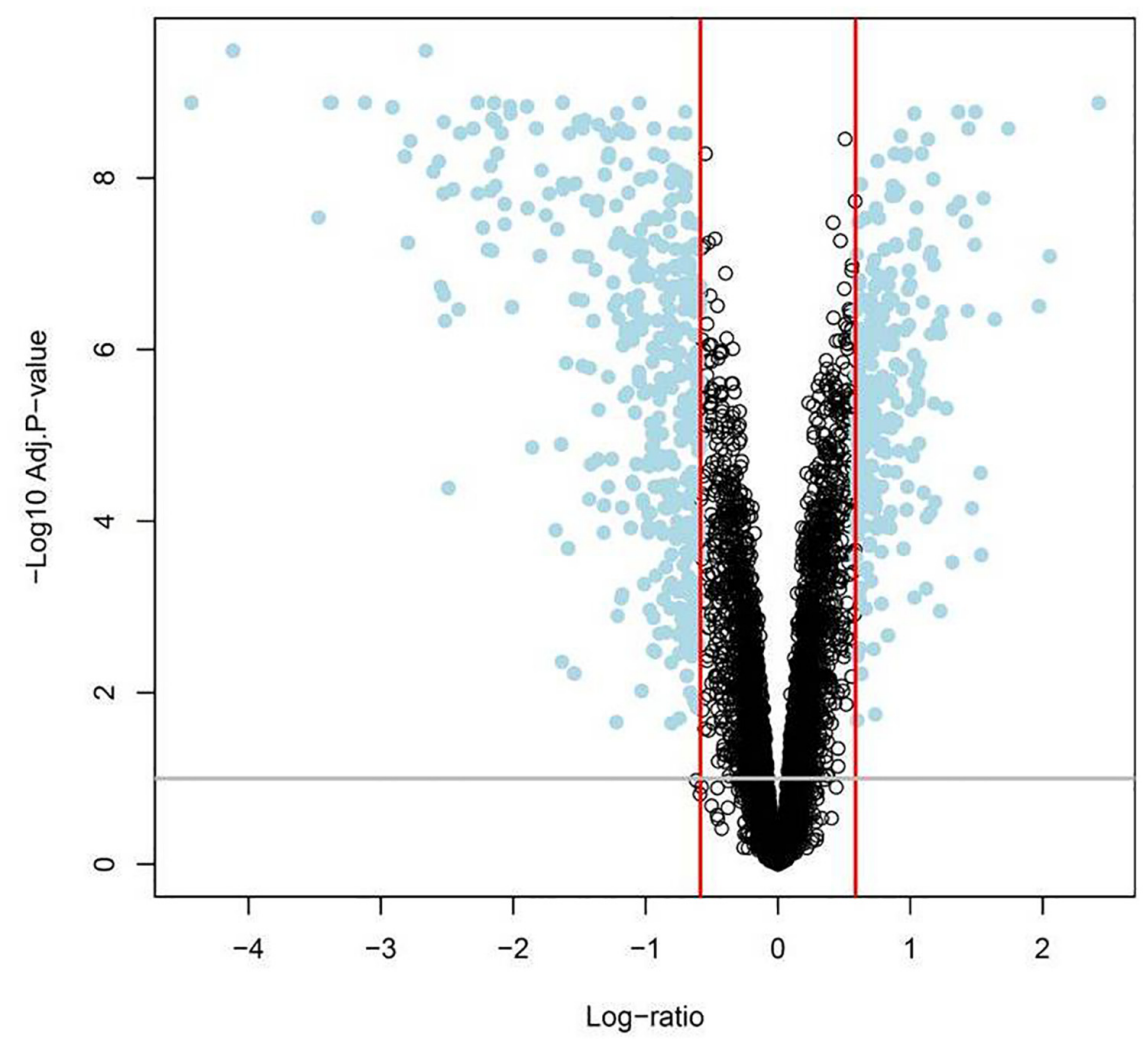
Pregnant women differential expression of visceral versus subcutaneous adipose tissue transcripts. A “volcano plot” shows the differential expression of all the transcripts tested in this comparison, with the log (base 10) of the FDR-adjusted probability values (q-value) (y-axis) plotted against the log (base 2) fold-changes (x-axis) between the visceral and subcutaneous adipose tissues.

**Fig 2:**
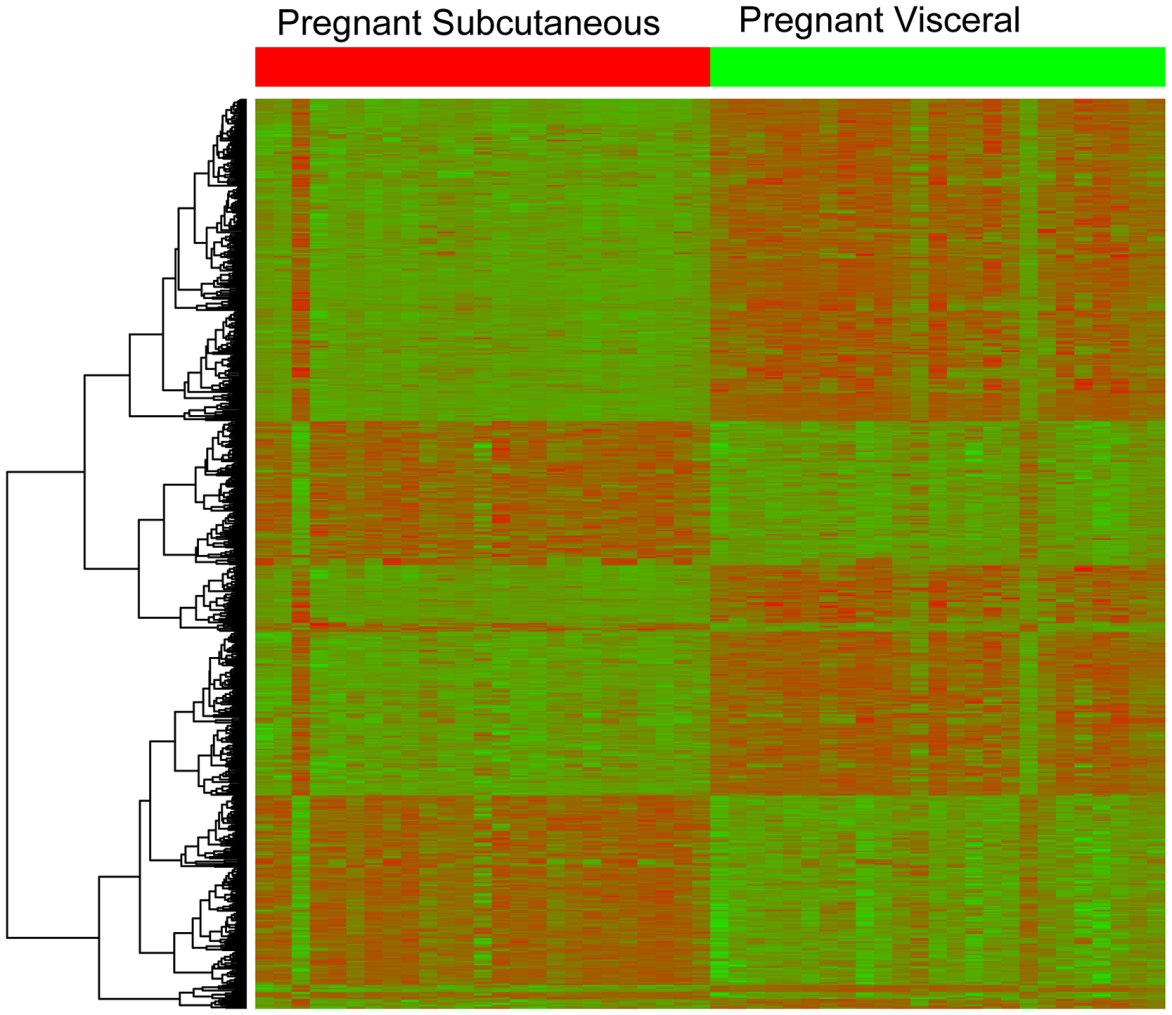
Heat map representing fat depot-specific differences in gene expression of pregnant women. The heat map uses a color scale to show the consistency of the expression levels within each group of samples as well as the differences between the groups that led to positive test results.

Microarray analysis demonstrated 644 transcripts that corresponded with 633 unique known genes that were differentially expressed between visceral and subcutaneous adipose tissue of pregnant women at term (q-value <0.1; fold change >1.5). A total of 391 genes had decreased expression, and 242 genes had increased expression in the subcutaneous, compared with visceral adipose tissue. A “volcano plot” shows the differential expression of all the transcripts tested in this comparison, with the log (base 10) of the FDR-adjusted probability values (q-value) (y-axis) plotted against the log (base 2) fold-changes (x-axis) between the visceral and subcutaneous adipose tissues (Fig 1). The heat map in Fig 2 uses a color scale to show the consistency of the expression levels within each group of samples as well as the differences between the groups that led to positive test results. A list of the top 10 differentially expressed transcripts between visceral and subcutaneous adipose tissues is presented in Table 2; the complete list of differentially expressed transcripts is available as supplementary material (S1 Table).

**Table 2:** A list of the top 10 differentially expressed transcripts between visceral and subcutaneous adipose tissues of pregnant women.

ENTREZ ID	SYMBOL	Gene Name	Fold Change*
55600	ITLN1	intelectin 1 (galactofuranose binding)	-21.60
9076	CLDN1	claudin 1	-17.33
3250	HP	haptoglobin	-11.08
93035	PKHD1L1	polycystic kidney and hepatic disease 1 (autosomal recessive)-like 1	-10.45
244	ANXA8	annexin A8	-10.35
642826	ANXA8L2	annexin A8-like 2	-8.68
246	ALOX15	arachidonate 15-lipoxygenase	-7.52
10950	CXADR	coxsackie virus and adenovirus receptor	-7.06
3240	HPR	haptoglobin-related protein	-6.94
100294156	C4B	complement component 4B (Chido blood group)	-6.86

(*) The fold change represents the number of times the average expression in one group is higher than the one in the other group. Positive values mean higher expression in subcutaneous compared to visceral tissues, while negative values represent higher expression in visceral compared to subcutaneous tissues. Genes are ranked by absolute fold change. All q-values < 0.01.

Gene ontology meta-analysis of the significantly up- and down-regulated genes was performed to identify gene ontology terms that were represented by the differentially expressed genes. In this analysis, 82 biological processes were enriched (q-value <0.05); the top 10 biological processes are presented in Table 3; the complete list of differentially expressed transcripts is available as supplementary material (S2 Table). Pathway analysis of the significant genes was undertaken with an overrepresentation method resulting in 12 KEGG pathways were significant (q-value <0.05) in the comparison between visceral and subcutaneous adipose tissues (Table 4); the complete list of differentially expressed transcripts is available as supplementary material (S3 Table). The five most significant pathways include: 1) the ECM-receptor interaction (S1 Fig); 2) the PPAR signaling pathway (S2 Fig); 3) protein digestion and absorption (S3 Fig); 4) focal adhesion; and 5) complement and coagulation cascades.

**Table 3:** A list of top 10 enriched biological processes in the comparison between visceral and subcutaneous adipose tissues of pregnant women.

Biological process	Genes in differentially expressed list, n	Genes in reference array, n	Odds Ratio	q-value
circulatory system development	85	657	3.43	0.000
multicellular organismal process	300	4412	2.15	0.000
localization of cell	83	765	2.77	0.000
response to wounding	86	905	2.37	0.000
retinal metabolic process	7	8	144.91	0.000
regulation of inflammatory response	19	103	4.83	0.000
multicellular organismal development	121	1894	1.80	0.000
cell adhesion	34	306	2.88	0.000
positive regulation of cellular component movement	28	212	3.23	0.000
locomotion	33	301	2.86	0.000

**Table 4:** A list of the 10 KEGG pathways that were significant in the comparison between visceral and subcutaneous adipose tissues of pregnant women.

Map Name	Genes in differentially expressed list, n	Genes in reference array, n	Odds Ratio	q-value
ECM-receptor interaction	16	72	5.55	0.000
PPAR signaling pathway	12	55	5.34	0.001
Protein digestion and absorption	13	64	4.89	0.001
Focal adhesion	23	171	3.05	0.001
Complement and coagulation cascades	11	49	5.52	0.001
Cytokine-cytokine receptor interaction	23	195	2.61	0.004
Arrhythmogenic right ventricular cardiomyopathy (ARVC)	11	63	4.02	0.007
Cell adhesion molecules (CAMs)	14	103	3.01	0.015
Malaria	8	40	4.71	0.015
Steroid hormone biosynthesis	6	28	5.11	0.039

#### Non-pregnant women: visceral versus subcutaneous

Microarray analysis demonstrated significant changes in the transcriptome of visceral and subcutaneous adipose tissues of non-pregnant women. In total, 226 unique genes were differentially expressed (q-value <0.1; fold-change >1.5). A total of 147 genes had decreased expression, and 79 genes had increased expression in the subcutaneous, compared with visceral, adipose tissue. A list of the top 10 differentially expressed genes between visceral and subcutaneous adipose tissues is presented in Table 5; the complete list of differentially expressed transcripts is available as supplementary material (S4 Table).

**Table 5:** A list of the top 10 differentially expressed transcripts between visceral and subcutaneous adipose tissues of non-pregnant women.

ENTREZ ID	SYMBOL	Name	Fold Change*	q-value
55600	ITLN1	intelectin 1 (galactofuranose binding)	-11.5	0.09
9076	CLDN1	claudin 1	-8.3	0.09
730	C7	complement component 7	-5.0	0.09
244	ANXA8	annexin A8	-5.0	0.09
93035	PKHD1L1	polycystic kidney and hepatic disease 1 (autosomal recessive)-like 1	-4.6	0.09
100294156	C4B	complement component 4B (Chido blood group)	-4.4	0.09
642826	ANXA8L2	annexin A8-like 2	-4.3	0.09
5999	RGS4	regulator of G-protein signaling 4	-4.1	0.09
246	ALOX15	arachidonate 15-lipoxygenase	-3.9	0.09
7980	TFPI2	tissue factor pathway inhibitor 2	-3.9	0.09

(*) The fold change represents the number of times the average expression in one group is higher than the one in the other group. Positive values mean higher expression in subcutaneous compared to visceral tissues, while negative values represent higher expression in visceral compared to subcutaneous tissues. Genes are ranked by absolute fold change.

Enrichment analyses identified 26 biological processes and five KEGG pathways that were significantly enriched in differentially expressed genes (q-value <0.05) in the comparison between visceral and subcutaneous adipose tissues. A list of the 10 enriched biological processes in the comparison between visceral and subcutaneous adipose tissues of non-pregnant women is presented in Table 6; the complete list is available as supplementary material (S5 Table). The significant pathways were: 1) complement and coagulation cascades; 2) *Staphylococcus aureus* infection; 3) prion diseases; 4) Chagas disease (American trypanosomiasis); and 5) retinol metabolism.

**Table 6:** A list of the 10 enriched biological processes in the comparison between visceral and subcutaneous adipose tissues of non-pregnant women.

Biological process	Genes in differentially expressed list, n	Genes in reference array, n	Odds Ration	q-value
complement activation, classical pathway	7	23	26.80	0.0001
protein activation cascade	8	45	13.37	0.001
cell adhesion	25	515	3.45	0.001
retinol metabolic process	5	14	33.68	0.002
retinal metabolic process	4	8	60.31	0.003
single-multicellular organism process	83	3703	2.02	0.004
localization of cell	30	765	2.68	0.004
circulatory system development	27	657	2.79	0.005
cellular response to cAMP	5	22	17.82	0.009
developmental process	59	2336	2.02	0.010

#### Pregnant versus non-pregnant women: subcutaneous adipose tissue

**Fig 3:**
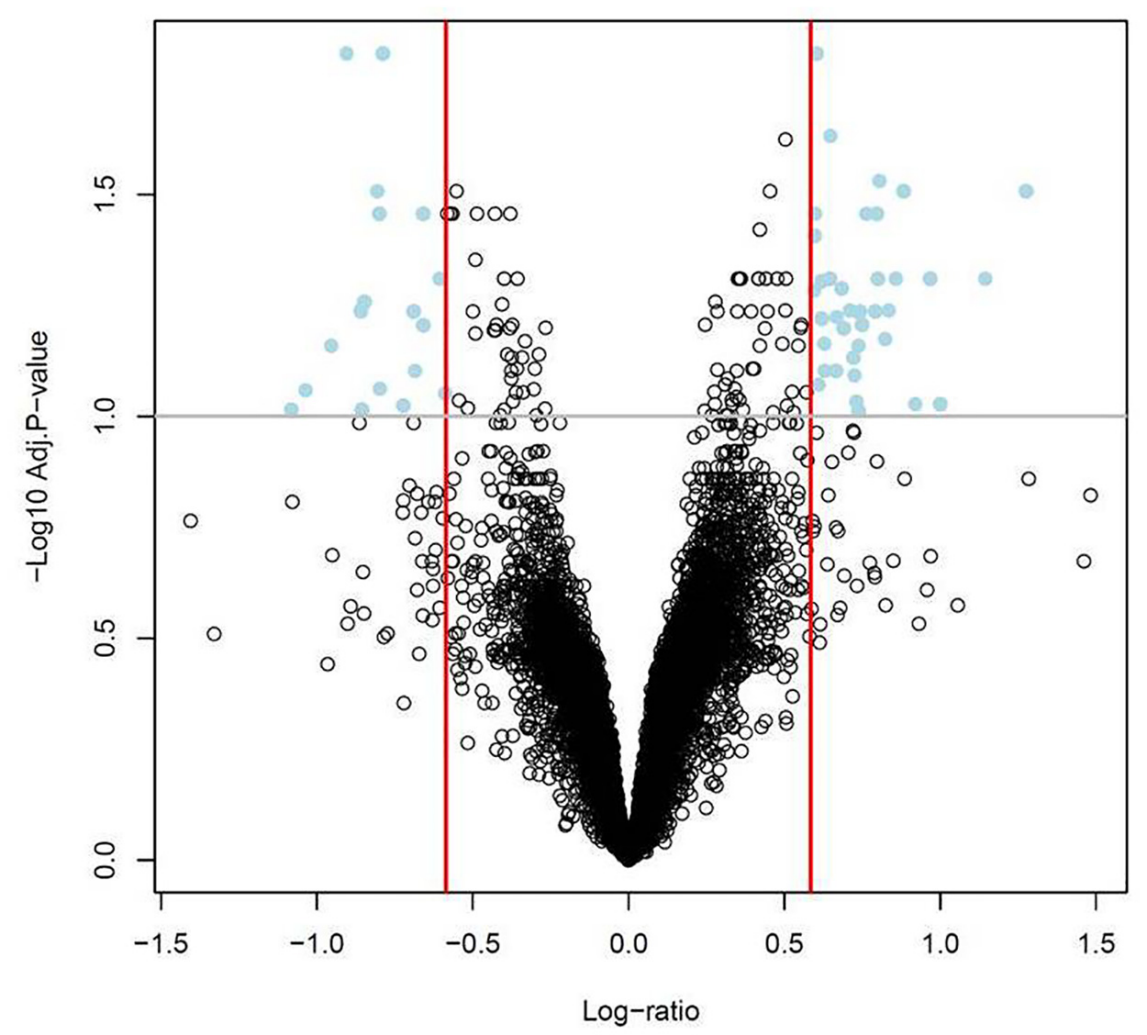
Differential expression of subcutaneous adipose tissue transcripts in pregnant vs. non-pregnant women. A “volcano plot” shows the differential expression of all tested transcripts, with the log (base 10) of the FDR-adjusted probability values (y-axis) plotted against the log (base 2) fold-changes (x-axis) between pregnant and non-pregnant groups.

**Fig 4:**
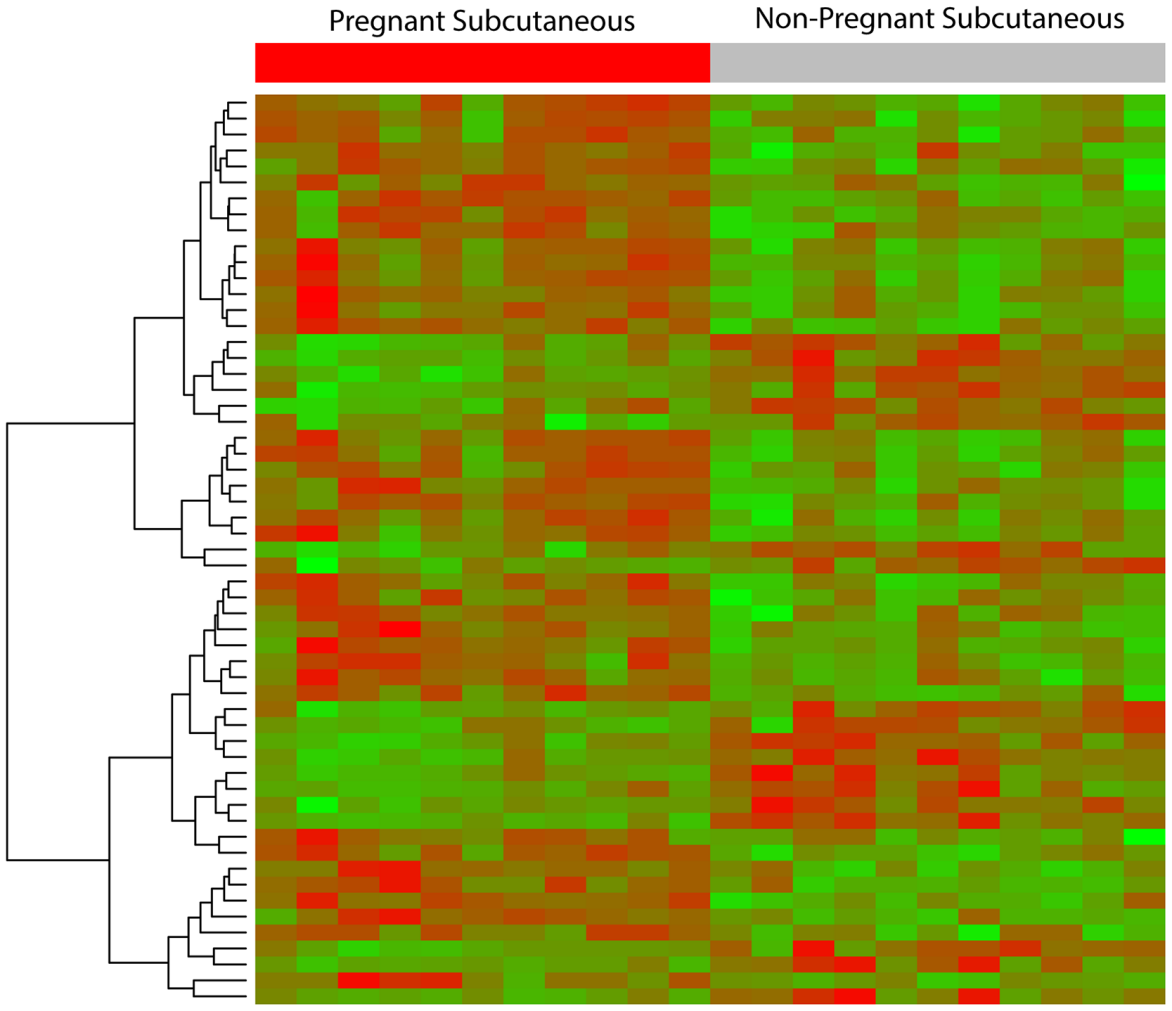
Heat map representing subcutaneous adipose tissue differences in gene expression of pregnant vs non-pregnant women. The heat map in Fig 4 uses a color scale to show the consistency of the expression levels within each group of samples as well as the differences between the groups that led to the positive test results.

Microarray analysis demonstrated significant changes in the transcriptome of subcutaneous adipose tissue between pregnant and non-pregnant women. In total, 57 transcripts corresponding to 56 known genes were differentially expressed (q-value <0.1; fold-change >1.5). A total of 19 genes had decreased expression, and 37 genes had increased expression in the subcutaneous adipose tissue of pregnant compared with non-pregnant women. A “volcano plot” shows the differential expression of all tested transcripts, with the log (base 10) of the FDR-adjusted probability values (y-axis) plotted against the log (base 2) fold-changes (x-axis) between pregnant and non-pregnant groups (Fig 3). The heat map in Fig 4 uses a color scale to show the consistency of the expression levels within each group of samples as well as the differences between the groups that led to the positive test results. A list of the top 10 differentially expressed transcripts in the subcutaneous adipose tissues between the pregnant and non-pregnant women is presented in Table 7; the complete list is available as supplementary material (S6 Table).

**Table 7:** A list of the 10 differentially expressed transcripts in the subcutaneous adipose tissues between the pregnant and non-pregnant women.

ENTREZ ID	SYMBOL	Gene Name	Fold Change*	q-value
729	C6	complement component 6	2.4	0.031
81617	CAB39L	calcium binding protein 39-like	2.2	0.049
81575	APOLD1	apolipoprotein L domain containing 1	-2.1	0.097
390075	OR52N5	olfactory receptor, family 52, subfamily N, member 5	-2.1	0.087
643616	MOP-1	MOP-1	2.0	0.094
11326	VSIG4	V-set and immunoglobulin domain containing 4	2.0	0.049
79473	OR52N1	olfactory receptor, family 52, subfamily N, member 1	-1.9	0.069
51338	MS4A4A	membrane-spanning 4-domains, subfamily A, member 4	1.9	0.094
3099	HK2	hexokinase 2	-1.9	0.015
10149	GPR64	G protein-coupled receptor 64	1.8	0.031

(*) The fold change represents the number of times the average expression in one group is higher than the one in the other group. Positive values mean higher expression in subcutaneous tissues of pregnant compared to non-pregnant women, while negative values represent higher expression subcutaneous tissues of non-pregnant women compared to pregnant women. Genes are ranked by absolute fold change.

Gene ontology meta-analysis revealed four biological processes that were enriched at q-value <0.05: 1) complement activation, classical pathway; 2) protein activation cascade; 3) immunoglobulin-mediated immune response; and 4) humoral immune response. Pathway analysis identified two KEGG pathways that were significantly enriched (q-value,<0.05) in the comparison between the subcutaneous adipose tissue samples of pregnant and non-pregnant women: 1) complement and coagulation cascades (S4 Fig), and 2) Prion diseases (S5 Fig).

#### Pregnant versus non-pregnant women: visceral adipose tissue

Microarray analysis demonstrated significant changes in the transcriptome of visceral adipose tissue between pregnant and non-pregnant women. In total, three unique genes had increased expression in the visceral adipose tissue of pregnant compared with non-pregnant women (Table 8).

**Table 8:** A list of the 3 differentially expressed transcripts in the visceral adipose tissues between the pregnant and non-pregnant women.

SYMBOL	ENTREZ ID	Name	Fold Change	q-value
CA1	759	carbonic anhydrase I	2.4	0.07
GPR64	10149	G protein-coupled receptor 64	2.0	0.05
CXorf21	80231	chromosome X open reading frame 21	1.5	0.05

#### Alternative splicing

The Affymetrix Human Exon 1.0 ST array that we used in this study allowed us to test for differential exon usage (a.k.a. differential/alternative splicing) in addition to the differential expression analysis described above. Significant differences in exon usage rates were found between visceral and subcutaneous adipose tissues of pregnant women and between pregnant and non-pregnant women in the subcutaneous region. Forty-two alternative splicing events in 36 unique genes were associated with the regional differences of the adipose tissue of pregnant women. For six of the 36 affected genes, the evidence for differential splicing was found for two distinct Affymetrix probesets that either targeted the same exon of the gene [peptidase domain containing associated with muscle regeneration 1 (*PAMR1*) and serine/arginine repetitive matrix 2 (*SRRM2*)] or different exons of the same gene [kinase non-catalytic C-lobe domain (KIND) containing 1 (*KNDC1*), the podocalyxin-like (*PODXL*), solute carrier family 7 (cationic amino acid transporter, y+ system), member 8 (*SLC7A8*), and desmin (*DES*)]. A list of the top 10 alternative splicing events associated with the regional differences of the adipose tissue of pregnant women is presented in Table 9; the complete list is available as supplementary material (S7 Table). Fifty percent of the genes that were affected by alternative splicing were also differentially expressed.

**Table 9:** A list of the alternative splicing events associated with the regional differences of the adipose tissue of pregnant women.

Symbol	Gene Name	Transcript ID	Exon ID	Probeset ID	Diff. mean FIRMA	q-value
ABLIM1	actin binding LIM protein 1	3307939	619043	3308001	2.851	0.000
ADRA2C	adrenergic, alpha-2C-, receptor	2716328	249900	2716338	-2.288	0.004
CRB2 ^#^	crumbs homolog 2 (Drosophila)	3188478	544486	3188501	-2.842	0.000
DAPK1 ^#^	death-associated protein kinase 1	3177880	538110	3177903	3.061	0.000
DCLK1 ^#^	doublecortin-like kinase 1	3509473	742860	3509602	-3.042	0.000
DES*	desmin	2528476	131889	2528483	-2.660	0.007
DES*	desmin	2528476	131895	2528491	-2.291	0.003
FAIM3	Fas apoptotic inhibitory molecule 3	2452977	84252	2452981	-2.944	0.000
GATA6 ^#^	GATA binding protein 6	3781245	908232	3781284	-2.220	0.000
GFPT2^#^	glutamine-fructose-6-phosphate transaminase 2	2890660	359171	2890703	2.247	0.000

*Genes showing differential exon usage for two exons

^#^ Genes demonstrating differential exon usage that were also differentially expressed

**Fig 5:**
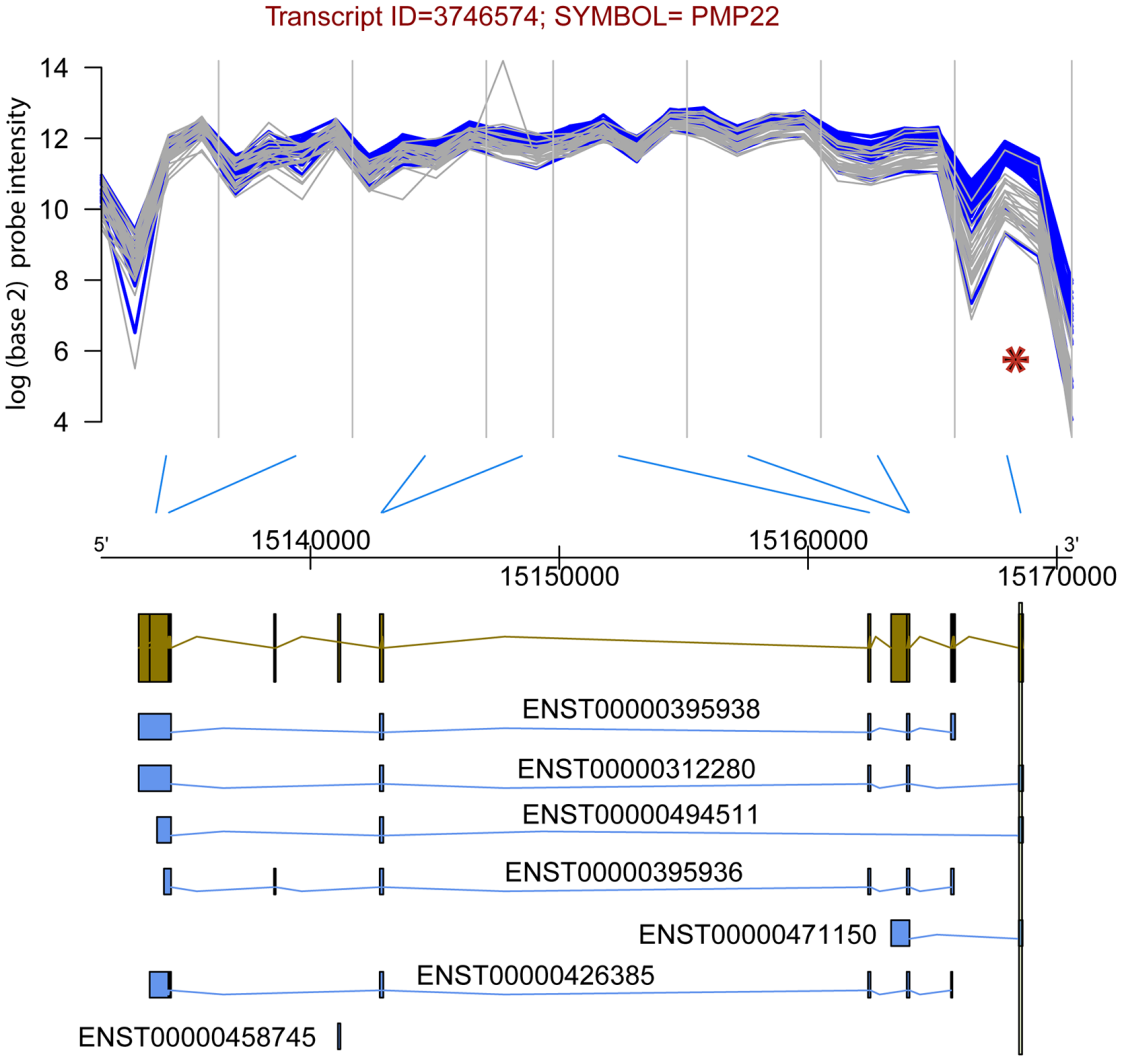
Normalized probe expression data against the genomic coordinates for gene PMP22. The figure shows the normalized probe intensity for gene PMP22 plotted as a function of the genomic coordinates from the 5’ to the 3’ end. Each line corresponds to one sample (blue: subcutaneous and grey: visceral). The probesets, each containing 4 probes, are separated by vertical grey lines. The ideogram under the genomic axis shown in gold color represents the gene model, with each vertical rectangle denoting one exon. Under the gene model are depicted known ENSEMBL database transcripts (in blue) that either include or exclude the exon that shows differential usage between groups, and which is highlighted with a vertical rectangle across all ideograms. For most probes used to target this gene, the expression level is about the same in the visceral and subcutaneous samples, except for the probes targeting the last exon for which the expression in the subcutaneous (blue) group, the expression is consistently higher.

For an illustration of the differential exon usage between the visceral and subcutaneous adipose tissues of pregnant women see Fig 5 which depicts the normalized probe expression data against the genomic coordinates for gene PMP22. The figure shows the normalized probe intensity for gene PMP22 plotted as a function of the genomic coordinates from the 5’ to the 3’ end. Each line corresponds to one sample (blue: subcutaneous and grey: visceral). The probesets, each containing 4 probes, are separated by vertical grey lines. The ideogram under the genomic axis shown in gold color represents the gene model, with each vertical rectangle denoting one exon. Under the gene model are depicted known ENSEMBL database transcripts (in blue) that either include or exclude the exon that shows differential usage between groups, and which is highlighted with a vertical rectangle across all ideograms. For most probes used to target this gene, the expression level is about the same in the visceral and subcutaneous samples, except for the probes targeting the last exon for which the expression in the subcutaneous (blue) group, the expression is consistently higher. As a consequence the FIRMA scores, indicative of the exon usage, are significantly higher in the subcutaneous than in the visceral samples.]

Differential splicing associated with labor was found for three genes in the subcutaneous adipose tissue, namely LIMS1 (LIM and senescent cell antigen-like domains 1), YY1AP1 (YY1 associated protein 1) and GSTK1 (glutathione S-transferase kappa 1). S6 Fig (see legend of Fig 5 for details) shows that for one of the middle exons of the gene GSTK1, the usage rate is lower in the labor group than in the not in labor group, while this is not the case for any other of the exons. Such a phenomenon can be explain for instance by an imbalance in the abundance of the isophorm that includes the exon (ENSEMBLE ID: ENST00000479303) and the remaining ones documented in the ENSEMBLE database which skip this exon.

We did not find significant differences in exon usage rates between pregnant and non- pregnant women in the visceral adipose tissue.

## Discussion

### Visceral versus subcutaneous adipose tissue in pregnant women

To our knowledge, this is the first study that describes the transcriptome of visceral and subcutaneous adipose tissues in pregnant women. Previous reports regarding gene expression in adipose tissue of pregnant women have used a targeted approach.[171–189] High-dimensional biology techniques allow comprehensive and unbiased insight into complex physiologic events including the investigation of the reproductive tract.[190–202] Using high-throughput techniques, differential gene and protein expressions have been reported in pregnant women in the uterine cervix,[203–211] human myometrium,[212–218] chorioamniotic membranes,[219,220] amniotic fluid,[221–230] and umbilical cord blood.[231] While region-specific differences were extensively investigated in non-pregnant individuals using both targeted and high-dimensional biology techniques,[118–120,128,130,131,149–151,232–253] the comprehensive gene expression, biological processes, and pathways associated with gestational adiposity have not yet been described. We used an unbiased approach to characterize the transcriptome of visceral and subcutaneous adipose tissues in pregnant and non-pregnant women to gain an understanding of pregnancy-related global changes in adipose tissue depot-specific gene expression and splicing.

The findings of the present study provide evidence for pregnancy-associated differences between visceral and subcutaneous adipose tissues. Compared with non-pregnant women, the magnitude of regional differences in the transcriptome of pregnant women was larger, with the fold changes of the top ten genes ranging from 6.9 to 21.6 in pregnant women compared to 4.6 to 11.5 in non-pregnant women. These findings suggest that adipose tissue depot-specific differences in gene expression are more accentuated in human gestation. Remarkably, among the top five differentially expressed genes between visceral and subcutaneous adipose tissues, four are common for both pregnant and non-pregnant women. These genes encode for omentin (intelectin 1), claudin 1, polycystic kidney and hepatic disease 1 (autosomal recessive)-like 1, and annexin A8. Consistent with this finding, among the top 100 differentially expressed genes between visceral and subcutaneous adipose tissues, 53 are common for both pregnant and non-pregnant women, suggesting that many of the depot-specific alterations are common for these groups yet the magnitude of changes is higher in pregnant women.

Omentin (also named intelectin) is a secretory protein that has been recently identified as a new depot-specific adipokine.[254–256] Several lines of evidence support the highly selective expression of omentin in human visceral adipose tissue: 1) omentin mRNA was predominantly expressed in visceral compared with subcutaneous fat;[255,257–259] 2) qRT-PCR demonstrated that omentin mRNA was expressed in stromal vascular cells, isolated from omental adipose tissue, with more than 150-fold less in subcutaneous cell fractions;[255] and 3) consistent with these findings, omentin was detected in a culture medium of omental, but not of subcutaneous, fat explants.[255] Importantly, qRT-PCR has demonstrated that omentin is expressed in stromal vascular cells but not in adipocytes.[255] Our results confirm that there is a significant over-expression of omentin in visceral compared to subcutaneous adipose tissue. We were able to extend the abovementioned reports by demonstrating over-expression of this adipokine in visceral fat of pregnant women. Furthermore, while the fold-change increase of omentin expression in visceral adipose tissue was 11.5 in the non-pregnant state, during pregnancy the fold-change was approximately twice as high (21.6), suggesting that this adipokine may play a role in the metabolic adaptations of visceral fat to normal gestation.

Omentin enhances insulin-stimulated glucose uptake in human adipocytes and triggers AKT signaling.[255,256] This adipokine does not stimulate basal glucose transport on its own, indicating that omentin has no intrinsic insulin-mimic activity. Thus, it has been suggested that depot-dependent insulin action is subject to modulation by this adipokine. Omentin has been detected in maternal circulation;[188,260,261] however, how omentin concentrations change during pregnancy is not yet known. It can be speculated that the insulin-sensitizing effect of this insulin-sensitizing hormone may be of special importance during pregnancy in order to balance the "diabetogenic" effect of several placental hormones. Alterations in omentin expression and/or secretion may also account for the association between maternal obesity and complications of pregnancy. Indeed, pre-existing maternal obesity is associated with lower omentin-1 expression in adipose tissue and maternal plasma.[188] In conclusion, the dramatic over-expression of omentin in visceral adipose tissue during pregnancy may point to a regulatory role of this adipokine in the depot-dependent insulin action that may alleviate the pregnancy-related insulin resistance.

Gene ontology and pathway analysis revealed functional categories associated with the adipose tissue depot-specific expression changes in human pregnancy. Specifically, the biological process related to extracellular matrix–receptor interactions, inflammation, metabolism, and tissue development characterized the pregnancy-related adipose tissue depot-specific alterations. The extracellular matrix–receptor interaction and PPAR signaling pathway were among the most impacted signaling pathways in the visceral adipose tissue of pregnant women. Interestingly, the extracellular matrix–receptor interaction pathway was also the most impacted signaling pathway at the site of rupture in the chorionic membranes[220] and has been implicated in cervical ripening before the onset of labor at term in human pregnancy.[205] Peroxisome proliferator-activated receptors (PPARs) are nuclear hormone receptors that are activated by fatty acids and their derivatives. PPARs are abundantly expressed in adipose tissue and central to the regulation of pre-adipocyte differentiation through transcriptional control of adipocyte-specific genes.[234,262,263] PPARs include three subtypes: PPAR -α, -β/δ, and -γ, and each is encoded in a separate gene.[264] PPAR-γ is considered the master adipogenic regulator,[263,265] and it has an essential role in maintaining mature adipocyte function.[265–267] This is of special importance as human pregnancy is characterized by increased accumulation of visceral fat. Indeed, using longitudinal ultrasound measurements, a significant increase in intraabdominal to subcutaneous ratio was observed during the third trimester when compared with the first and second trimesters.[268] Accordingly, parity is associated with increased abdominal fat retention for months and years after delivery.[269–271] Taken together, our findings suggest that, during pregnancy, biological processes aimed at the expansion and development of both components of adipose tissue, i.e., the adipocytes and stromal vascular cells, are activated in visceral adipose fat. Thus, the results of this study may provide a putative molecular mechanism(s) by which pregnancy-associated increase and expansion of fat accrual occurs.

Bashiri et al[272] have determined alterations in genome-wide transcription expression in visceral and abdominal subcutaneous fat deposits in obese and lean pregnant women (4 in each group) using Affymetrix Human Exon 1.0 st platform. The authors reported that global alteration in gene expression was identified in pregnancy complicated by obesity and the identification of indolethylamine N-methyltransferase, tissue factor pathway inhibitor-2, and ephrin type-B receptor 6, that were not previously associated with fat metabolism during pregnancy. In addition, subcutaneous fat of obese pregnant women demonstrated increased coding protein transcripts associated with apoptosis as compared to lean pregnant women. Several of the top 36 candidate genes with the greatest variation in expression between subcutaneous and omental fat in both normal and obese pregnant women reported in the study of Bashiri et al.[272] were also found to be differentially expressed in the present study including NTNG1, KCNT2, LYPD6 and others.

### Pregnant versus non-pregnant states: gestational-related alterations in the subcutaneous and visceral adipose tissue transcriptome

To our knowledge, there has been no report comparing the visceral and subcutaneous adipose tissue transcriptome between pregnant and non-pregnant women. We report herein that 57 and three genes were differentially expressed between pregnant and non-pregnant women in subcutaneous and visceral adipose tissues, respectively. Intriguingly, the gene encoding G protein-coupled receptor 6 (*GPR64*) was over-expressed in both subcutaneous and visceral adipose tissues of pregnant women. GPR64 (also known as HE6—human epididymis-specific protein 6) is an orphan member of the LNB-TM7(B2) subfamily of G-protein-coupled receptors.[273–276] GPR64 mRNA is highly expressed in the epithelia of ductuli efferentes and proximal epididymis.[273–278] It has been suggested to function in the control of water balance and fluid reabsorption in the male excurrent ducts.[279–281] qRT-PCR studies with numerous tissue probes from mouse, rat, and human specimens as well as microarray analyses of essentially all human tissues and organs revealed a highly epididymis-restricted expression of HE6.[275] Thus, to our knowledge, this report represents the first evidence that GPR64 is expressed in human adipose tissue. The biological importance of GPR64 in adipose tissue, and specifically during gestation, has to be evaluated. HE6/GPR64 is an ‘orphan’ member of the adhesion GPCRs,[274–276] and an endogenous ligand(s) is presently unknown. Moreover, ligand prediction for GPCRs is extremely difficult since ligands for GPCRs are associated with remarkable variation.[275]

The findings of this study indicate that three out of four biological processes enriched in subcutaneous fat during pregnancy (i.e. complement activation, classical pathway, immunoglobulin mediated immune response and humoral immune response) are related to inflammation. This finding is consistent with a large body of evidence indicating that adipose tissue can orchestrate an inflammatory response, including: 1) knockout mice for IL-6,[282] TNF-α,[283] PAI-1,[284] IL-18,[285] IL-1α,[286] MCP-1,[287] and JNK1[288] are often obese or have a metabolic phenotype related to obesity; 2) adipose tissue is an important site for the production of inflammatory mediators including TNF-α,[13,289–291] IL-6,[292–294] monocyte chemoattractant protein (MCP)-1,[295,296] C-reactive protein (CRP),[23,24,293,297–299] serum amyloid A,[300] and plasminogen activator inhibitor-1 (PAI-1);[301] 3) adipocytokines such as resistin,[302–305] visfatin,[306–308] and adipsin[309] have been implicated in the regulation of the innate immune responses, and leptin[310–315] and adiponectin[316–322] are involved in the regulation of both innate and adaptive limbs of the immune system; and 4) obesity is associated with high-circulating pro-inflammatory and acute phase reactant adipocytokines such as TNF-α,[323] IL-6,[294] and CRP.[293] Normal pregnancy is considered a pro-inflammatory state. The total white blood cell count in maternal blood increases with advancing gestational age and leukocytes derived from normal pregnant women are phenotypically and metabolically activated.[324,325] During pregnancy, there is also an increased circulating concentration of acute phase proteins. Of note, a growing body of evidence suggests that normal gestation is characterized by adipose tissue inflammation,[326]and this process seems to be accentuated toward the end of the pregnancy[327,328]. Collectively, our data suggest that pregnancy-related subcutaneous adipose inflammation may contribute to the generalized pro-inflammatory state that characterizes human gestation.

Resi V et al[329] have recently reported the results of a longitudinal study in which adipose tissue biopsies were obtained from the subcutaneous gluteal depot of healthy non-obese women who are in early (8–12 weeks of gestation) and late (36–38 weeks of gestation) pregnancy. Specimens obtained via liposuction were subjected to histologic examination and gene expression analysis using DNA microarray. The findings of our study are in agreement with the above-mentioned report[329] in which approximately 40% of pregnancy-associated changes were related to mediators of the immune response, extracellular matrix components.[329] In addition, comparison between pre-conception and early pregnancy gene expression revealed marked changes in genes regulating pathways for the inflammatory response and metabolism.[329] In contrast to the report by Resi V et al,[329] in which 26% of pregnancy-associated changes were related to lipid metabolism, we did not identify this biological process as significantly enriched in genes differentially expressed between pregnant and non-pregnant women. Several explanations can account for this discrepancy: 1) the methods by which specimens were obtained were different between the studies; and gene expression, as determined by microarray experiments, has been shown to be affected by the adipose tissue biopsy technique;[245] 2) subcutaneous adipose tissue gene expression varies as a function of the specific region from which specimens were obtained and 3) the microarray platforms used were different. A recent study has shown that a total of 2,890 transcripts were differentially expressed between four subcutaneous adipose depots: upper abdomen, lower abdomen, flank, and hip in normal weight women;[330] and 3) it has been proposed that gluteal and femoral adipocytes serve as energy stores during pregnancy and lactation to meet the increased need for energy during that time.[269,331] This view is supported by studies in humans demonstrating that the activity of lipoprotein lipase in adipocytes from the femoral region increases during pregnancy, whereas such a pattern was not detected for abdominal adipocytes.[331] Thus, different gene expression patterns among subcutaneous adipose depots (e.g. abdominal versus gluteal) may represent their diverse function during normal human pregnancy.

### Alternative splicing: a novel pregnancy-associated mechanism for the regional differences between visceral and subcutaneous adipose tissues

Alternative splicing of mRNA transcripts is the process by which cells can selectively include or exclude different sections of pre-mRNA during RNA processing.[332] Once translated, these altered transcripts result in closely related proteins expressed from a single locus.[332,332,333] Alternative splicing is a major biological process by which a relatively limited number of genes can be expended into elaborate proteomes.[334] It has been estimated that approximately two-thirds to three-quarters of all human genes undergo alternative splicing.[334–337] The splicing process may affect function, localization, binding properties, and stability of the encoded proteins.[334,338] Moreover, alternative splicing can also lead to degradation of the transcript.[334,339,340] It is an important regulatory mechanism that has been shown to be involved in several molecular pathways including angiogenesis and differentiation.[332,341]

To the best of our knowledge, this is the first report implicating alternative splicing in regional differences of adipose tissue either in pregnant or non-pregnant individuals. We identified 42 exons in 36 genes showing differential usage in the comparison between visceral and subcutaneous adipose tissues. Of note, significant results were found only for the comparison between adipose tissue depots of pregnant women, but not for non-pregnant individuals. This finding characterized pregnancy as a unique physiologic condition in which alternative splicing may account for the different biological functions of visceral versus subcutaneous adipose tissue. It can be postulated that the remodeling and adaptations of adipose tissue during gestation require a larger repertoire of proteins that can be achieved by alternative splicing.

### Strengths and limitations of the study

The major strengths of this study are novel findings reported herein, the employment of a high throughput technique in the investigation of adipose tissue during pregnancy, the evaluation of paired specimens, and the inclusion of well-matched, non-pregnant controls.

This report represents the first description of the transcriptome of adipose tissue visceral and subcutaneous transcriptome in pregnant women and the comparison between pregnant and non-pregnant gene expression in these fat depots. Using the Illumina GeneChip Human Exon 1.0 ST array, we analyzed exon level expression data to determine differential usage associated with adipose tissue regions and pregnancy. Significant differences in exon usage were found between visceral and subcutaneous adipose tissues of pregnant women, implicating alternative splicing in regional differences in adipose tissue for the first time. We have identified novel genes previously unrecognized to be differentially expressed in visceral versus subcutaneous adipose tissues. Furthermore, we have demonstrated the expression of G protein-coupled receptor 6 (GPR64) in both visceral and subcutaneous. This gene was thought to be expressed exclusively and abundantly in epithelia of ductuli efferentes and proximal epididymis. Several limitations of our study should be acknowledged, and these include the racial polarity of our patient population. As the study population consists mainly of African-American women, the generalization of our findings to pregnant women of different ethnic origins will require future investigation. In addition, this study was specifically designed to delineate differences between visceral and abdominal subcutaneous adipose tissues. Thus, adipose tissues of other regions were not evaluated. Finally, we recognize that the cross-section natural of this study does not allow us to demonstrate neither a temporal nor a causal relationship between gestation and alterations in adipose tissue region-specific gene expression.

In conclusion, we have provided evidence that the adipose tissue region-specific alterations in gene expression established in non-pregnant individuals are enhanced during human gestation. Furthermore, unique pregnancy-related gene expression characterized both visceral and especially subcutaneous adipose tissues. Finally, alternative splicing has been implicated in regional differences in adipose tissue for the first time. Collectively, these novel findings may provide a molecular mechanism for the pregnancy-related adipose tissue remodeling, expansion, metabolic adaptations and the inflammatory response.

## Supporting Information

S1 Fig Rendering of the ECM-receptor interaction KEGG pathway (hsa04512) showing genes and genes complexes as colored rectangles (blue = down-regulation, magenta = up-regulation) between subcutaneous and visceral tissues of pregnant women. The genes COL4A1, COL4A2, COL5A2 and COL3A1 are shown as a single rectangle (Collagen). The same is true about LAMB1 and LAMB3, which are represented as the Laminin rectangle.
(TIF)

S2 Fig Rendering of the PPAR signaling KEGG pathway (hsa03320) showing genes and genes complexes as colored rectangles (blue = down-regulation, magenta = up-regulation) between subcutaneous and visceral tissues of pregnant women. (TIF)

S3 Fig Rendering of the Protein digestion and absorption KEGG pathway (hsa04974) showing genes and genes complexes as colored rectangles (blue = down-regulation, magenta = up-regulation) between subcutaneous and visceral tissues of pregnant women. The genes COL12A1, COL15A1, COL3A1, COL4A1, COL4A2 and COL5A2 are shown as a single rectangle (Collagen). The same is true about ACE2, DPP4, MME and XPNPEP2, which are represented as the Peptidase rectangle.
(TIF)

S4 Fig Rendering of the Complement and coagulation cascades KEGG pathway (hsa04610) showing genes and genes complexes as colored rectangles (blue = down-regulation, magenta = up-regulation) between subcutaneous tissues of pregnant and non-pregnant women. The genes C1QA, C1QB and C1QC are shown as a single rectangle (C1Q).
(TIF)

S5 Fig Rendering of the Prion diseases KEGG pathway (hsa05020) showing genes and genes complexes as colored rectangles (blue = down-regulation, magenta = up-regulation) between subcutaneous tissues of pregnant and non-pregnant women. The genes C1QA, C1QB and C1QC are shown as a single rectangle (C1q).
(TIF)

S6 Fig Normalized probe expression data against the genomic coordinates for gene GSTK1. (TIF)

S1 File Supplementary methods. (DOC)

S1 Table Subcutaneous vs. visceral: pregnant women. (XLS)

S2 Table Subcutaneous vs. visceral in pregnant women: enriched biological processes. (DOC)

S3 Table Subcutaneous vs. visceral in pregnant women: KEGG pathways. (DOC)

S4 Table Subcutaneous vs. visceral: non-pregnant women. (XLS)

S5 Table Subcutaneous vs. visceral in non-pregnant women: enriched biological processes. (DOC)

S6 Table Subcutaneous adipose in pregnant vs. non-pregnant women. (DOC)

S7 Table Alternative splicing. (DOC)
