# Supplementary figures and images for: Characterization of Visceral and Subcutaneous Adipose Tissue Transcriptome and Biological Pathways in Pregnant and Non-Pregnant Women: Evidence for Pregnancy-Related Regional-Specific Differences in Adipose Tissue
Source: PLoS One. 2015 Dec 4;10(12):e0143779. doi: 10.1371/journal.pone.0143779 (PMC4670118; doi:10.1371/journal.pone.0143779)

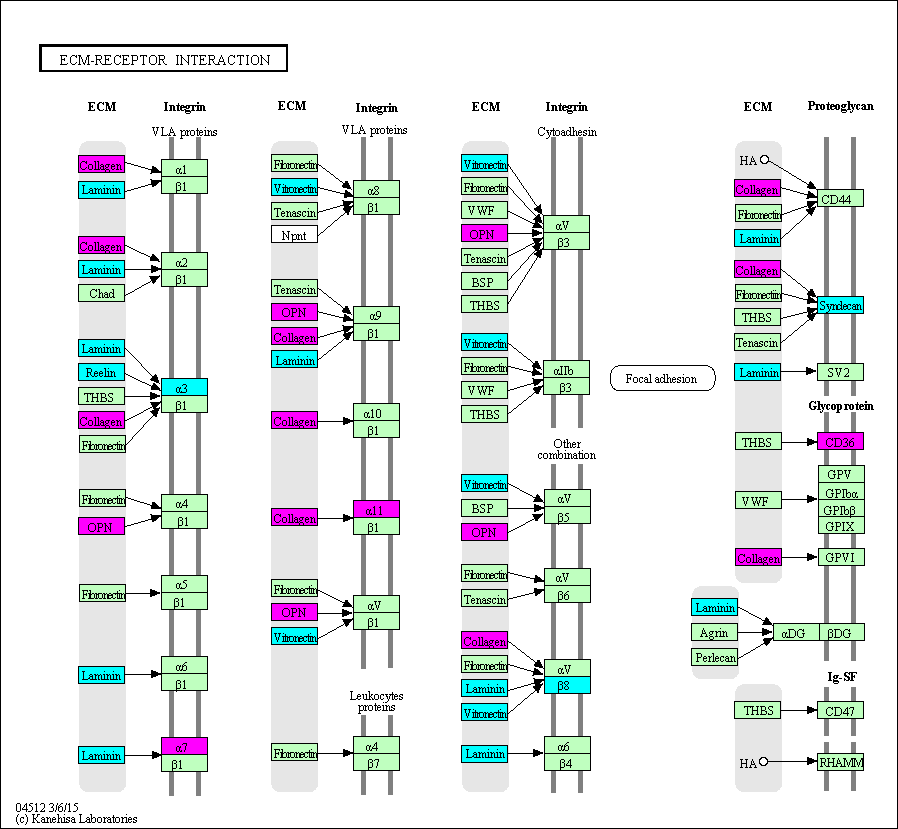

Supplement: S1 Fig — The genes COL4A1, COL4A2, COL5A2 and COL3A1 are shown as a single rectangle (Collagen). The same is true about LAMB1 and LAMB3, which are represented as the Laminin rectangle. (TIF) [file pone.0143779.s001.tif]

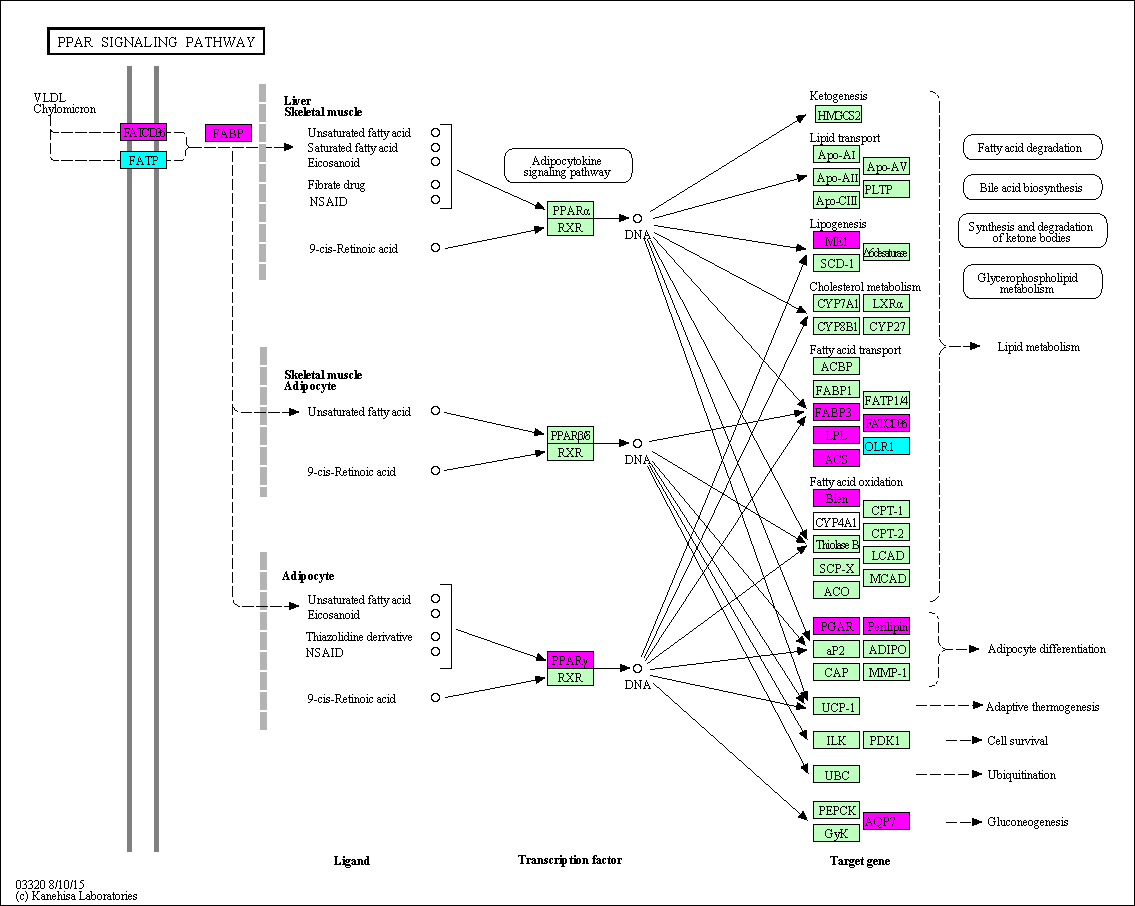

Supplement: S2 Fig — (TIF) [file pone.0143779.s002.tif]

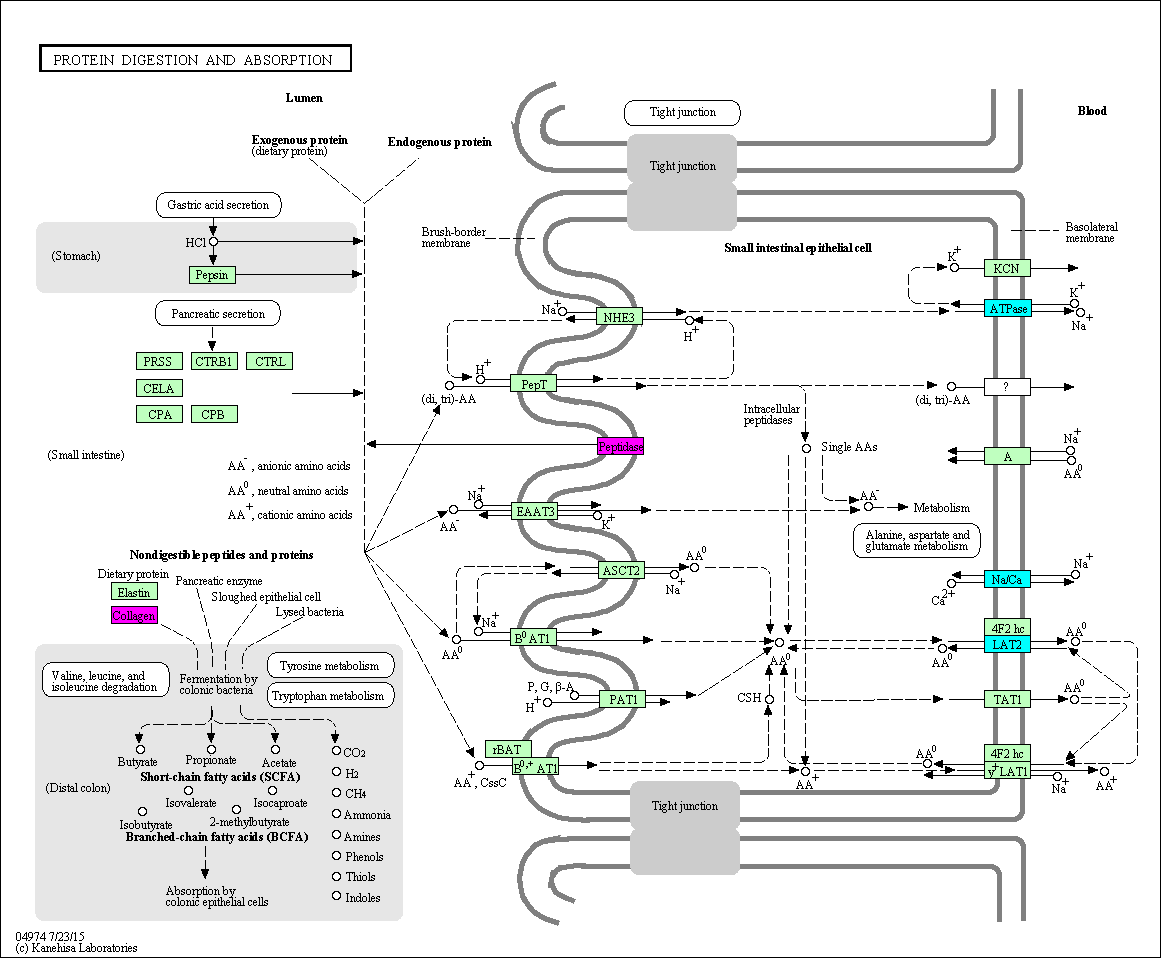

Supplement: S3 Fig — The genes COL12A1, COL15A1, COL3A1, COL4A1, COL4A2 and COL5A2 are shown as a single rectangle (Collagen). The same is true about ACE2, DPP4, MME and XPNPEP2, which are represented as the Peptidase rectangle. (TIF) [file pone.0143779.s003.tif]

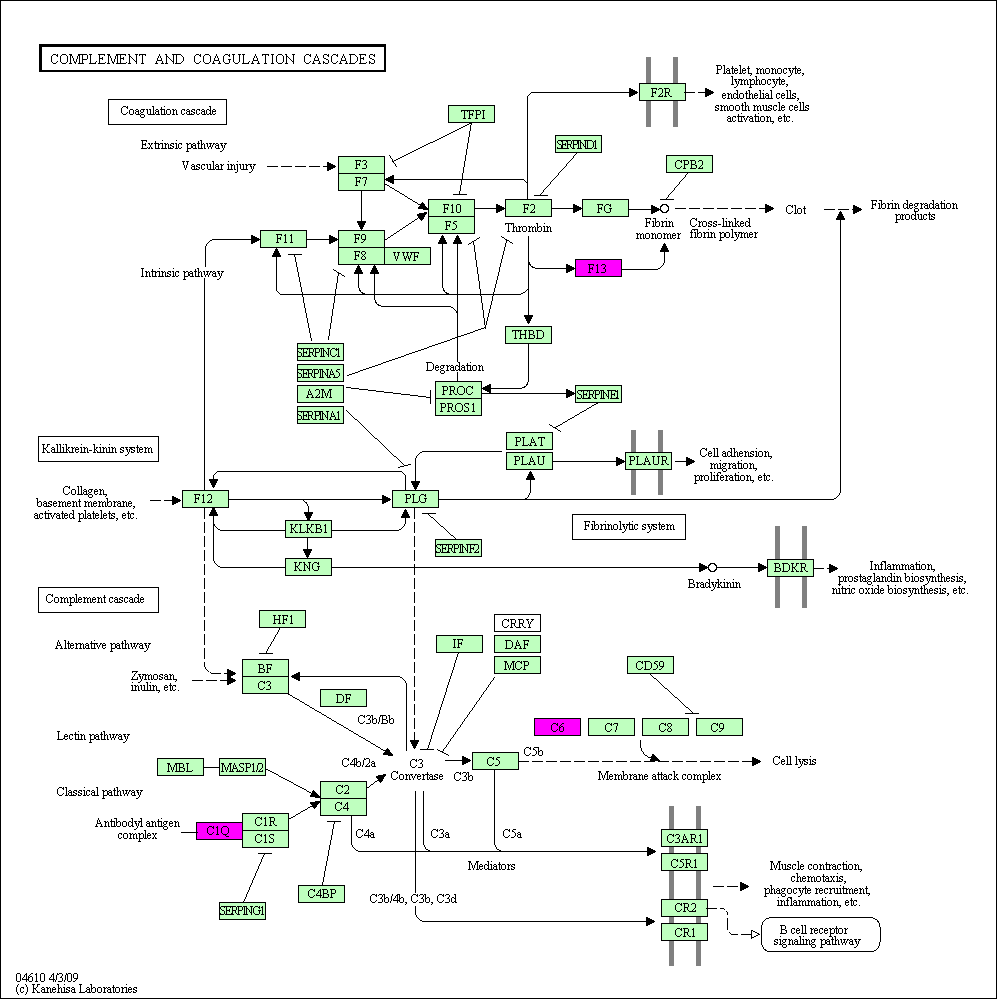

Supplement: S4 Fig — The genes C1QA, C1QB and C1QC are shown as a single rectangle (C1Q). (TIF) [file pone.0143779.s004.tif]

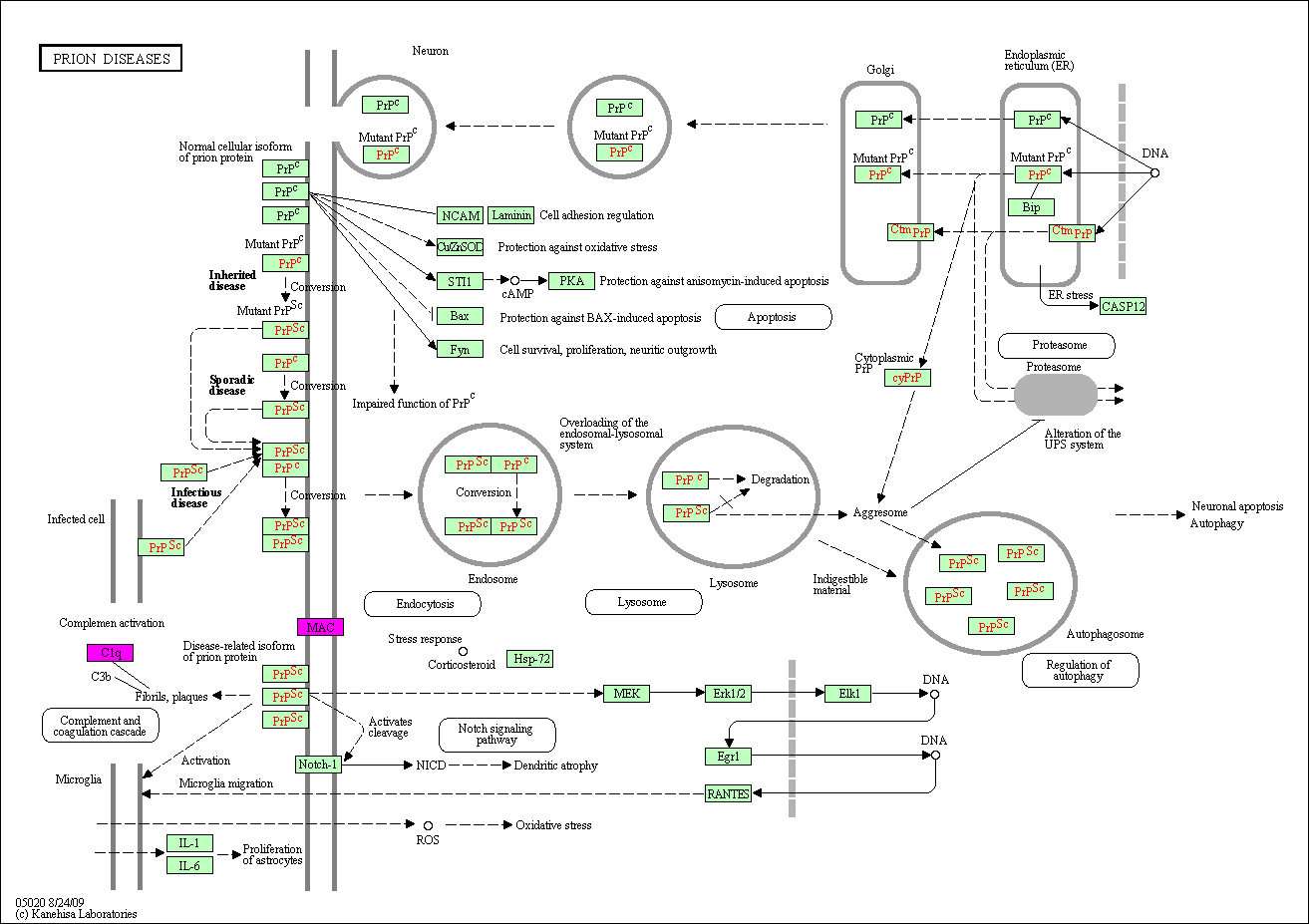

Supplement: S5 Fig — The genes C1QA, C1QB and C1QC are shown as a single rectangle (C1q). (TIF) [file pone.0143779.s005.tif]

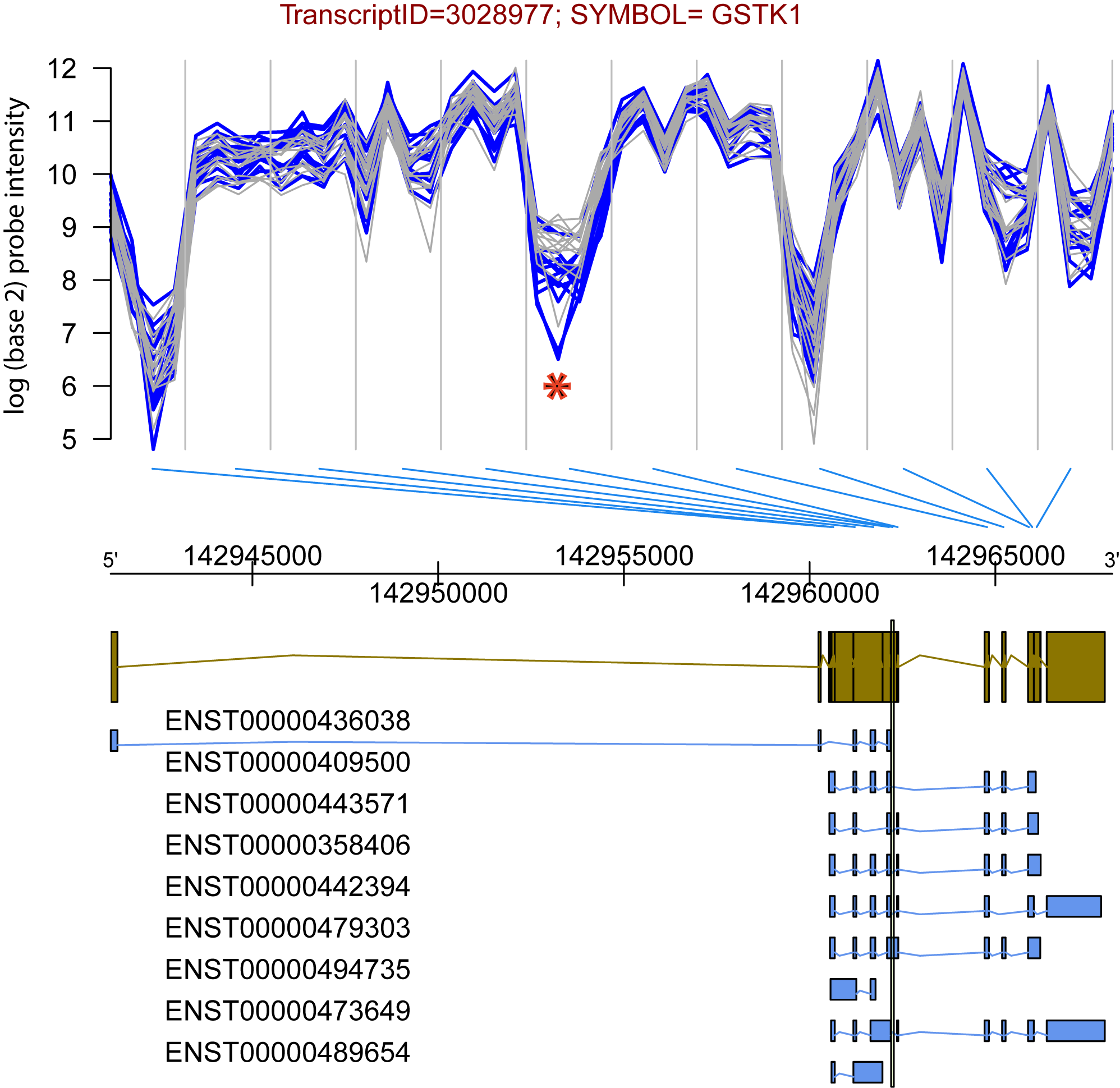

Supplement: S6 Fig — (TIF) [file pone.0143779.s006.tif]
