# Supplementary material for: Characterization of Visceral and Subcutaneous Adipose Tissue Transcriptome and Biological Pathways in Pregnant and Non-Pregnant Women: Evidence for Pregnancy-Related Regional-Specific Differences in Adipose Tissue
Source: PLoS One. 2015 Dec 4;10(12):e0143779. doi: 10.1371/journal.pone.0143779 (PMC4670118; doi:10.1371/journal.pone.0143779)
Supplement: S1 File — (DOC) [file pone.0143779.s007.doc]

**Exon array data analysis**

The GeneChip Human Exon 1.0 ST Array used in this study allowed us to determine the abundance of about one million exons based typically on one probeset per exon containing four probes. Exons are grouped into transcript clusters. The results presented in this article are based on the 18,708 core transcripts comprising 284,258 probesets for a total of 1,082,385 individual probes.

The goal of the differential expression analysis was to summarize the expression at transcript level and compare it between groups. The goal of the differential exon usage analysis is to detect exons whose departure from the overall transcript expression level differed between groups of samples.

1. **Differential expression**

The background correction and quantile normalization methods described [1] and implemented in the aroma.affymetrix package [2] were used to transform the raw probe intensity measurements in all samples. For differential expression analysis data was summarized at transcript level. A paired moderated [3] t-test was applied to test for differential gene expression between groups. Transcripts were selected as differentially expressed using combined criteria: False Discovery Rate (FDR) [4] adjusted p-values <10% and fold change in expression of at least 1.5. Only transcripts detected present in at least 5 samples were included in the analysis. The criteria for transcript detection are detailed below.

1. **Differential exon usage**
2. *Data preprocessing and filtering*

Summarization of probe intensity was performed at transcript level as well as at the exon/probeset level for this analysis using aroma.affymetrix package [2].

Exon/transcript pairs that had high potential for leading to false-positive results were removed by applying Affymetrix mandatory filtering [3] that involves discarding exons that were not deemed present or belonging to transcripts that were not deemed present. Exons and transcripts were called present in a given two-group comparison (e.g. pregnant vs. non-pregnant) as follows:

Present Exon: Consider all four probes (grouped in a probeset) used to target a given exon. Their intensity was compared to the median intensity of all probes on the array in the two groups of samples, and these probes were deemed present if they were above the median value. If at least three probes of the exon were present in a given sample, then the exon was deemed present in that sample. The exon was deemed present overall if it was present in at least one-half of the samples of either of the groups.

Present Transcript: Consider all probes that target a given transcript (targeting all exons of a given transcript cluster). If at least one-half of the probes of the transcript were present in a given sample, then the transcript was deemed present in that sample. The transcript was deemed present overall if it was present in at least one-half of the samples of each of the two groups.

We chose to incorporate additional filtering steps from secondary (suggested) and tertiary (optional) filtering described by Affymetrix as follows:

-discarding of probesets with high potential for cross-hybridization (as defined by Affymetrix);

-removal of genes that had very large expression differences between the two groups (above 10 fold); and,

-limiting the search to 18,708 high-confidence (core) units/transcript clusters based on Ref Seq transcripts and full-length mRNAs.

After all different types of filtering, and depending on the comparison of interest, about 45,000 exons were tested for differential usage.

*Testing for differential exon usage*

In order to identify differential exon usage between groups of samples, we used the FIRMA (Finding Isoforms Using Robust Multichip Analysis) method [4] to quantify how far (above or below) a given exon’s expression level was compared to the expected (average) transcript level in a given sample.

We applied a t-test for each probeset (typically one per exon) in each transcript based on the FIRMA scores, and found a difference in mean FIRMA scores between groups of two or more combined with a threshold of 0.1 on the False Discovery Rate adjusted p-values (called q-values) obtained from the nominal t-test p-values. This was a more stringent approach than described in another study [4] in which positive results were identified based only on the difference in mean FIRMA scores above 1.5 units.

*Data visualization*

Plotting of the probe-level expression data at exon levels vs. genomic coordinates was performed using functionality provided by the GenomeGraphs package. Known isoforms for a given gene were retrieved from the ENSEMBL database using the biomaRt package [5].

**References:**

1: Irizarry RA, Hobbs B, Collin F, Beazer-Barclay YD, Antonellis KJ, Scherf U, et al. Exploration, normalization, and summaries of high density oligonucleotide array probe level data. Biostatistics 2003;4:249-264.

2: Bengtsson H, K. Simpson,J. Bullard, K. Hansen aroma.affymetrix: A generic framework in R for analyzing small to very large Affymetrix data sets in bounded memory. Tech Report # 745 of the Department of Statistics, University of California, Berkeley February 2008.

3: Smyth GK, Yang YH, Speed T. Statistical issues in cDNA microarray data analysis. Methods Mol.Biol 2003;224:111-36.

4: Benjamini Y, Hochberg Y. Controlling the false discovery rate: a practical and powerful approach to multiple testing. J Royal Stat Soc B 1995;57:289-300.

5: Affymetrix Technical Note. Identifying and Validating Alternative Splicing Events.

6: Purdom E, Simpson KM, Robinson MD, Conboy JG, Lapuk AV, Speed TP FIRMA: a method for detection of alternative splicing from exon array data. Bioinformatics 2008;24:1707-1714.

:7 Durinck S, Moreau Y, Kasprzyk A, Davis S, De Moor B, Brazma A, et al. BioMart and Bioconductor: a powerful link between biological databases and microarray data analysis. Bioinformatics 2005;21:3439-3440
