# Supplementary material for: Characterization of Visceral and Subcutaneous Adipose Tissue Transcriptome and Biological Pathways in Pregnant and Non-Pregnant Women: Evidence for Pregnancy-Related Regional-Specific Differences in Adipose Tissue
Source: PLoS One. 2015 Dec 4;10(12):e0143779. doi: 10.1371/journal.pone.0143779 (PMC4670118; doi:10.1371/journal.pone.0143779)
Supplement: S2 Table — (DOC) [file pone.0143779.s009.doc]

**Table 3. A list of top 20 enriched biological processes in the comparison between visceral and subcutaneous adipose tissues of pregnant women**

| **q-value** | **Odds Ratio** | **Genes in reference array, n** | **Genes in differentially expressed list, n** | **Biological process** |
| --- | --- | --- | --- | --- |
| 0.000 | 3.43 | 657 | 85 | circulatory system development |
| 0.000 | 2.15 | 4412 | 300 | multicellular organismal process |
| 0.000 | 2.77 | 765 | 83 | localization of cell |
| 0.000 | 2.37 | 905 | 86 | response to wounding |
| 0.000 | 144.91 | 8 | 7 | retinal metabolic process |
| 0.000 | 4.83 | 103 | 19 | regulation of inflammatory response |
| 0.000 | 1.80 | 1894 | 121 | multicellular organismal development |
| 0.000 | 2.88 | 306 | 34 | cell adhesion |
| 0.000 | 3.23 | 212 | 28 | positive regulation of cellular component movement |
| 0.000 | 2.86 | 301 | 33 | locomotion |
| 0.000 | 3.17 | 215 | 28 | positive regulation of locomotion |
| 0.000 | 5.16 | 81 | 16 | positive regulation of angiogenesis |
| 0.000 | 2.41 | 467 | 45 | response to external stimulus |
| 0.000 | 3.32 | 193 | 26 | urogenital system development |
| 0.001 | 2.23 | 489 | 46 | regulation of body fluid levels |
| 0.002 | 3.90 | 117 | 18 | muscle organ development |
| 0.002 | 4.54 | 84 | 15 | neuron migration |
| 0.002 | 3.00 | 201 | 25 | reproductive system development |
| 0.003 | 1.89 | 838 | 66 | regulation of multicellular organismal development |
| 0.004 | 3.93 | 101 | 16 | positive regulation of epithelial cell proliferation |
