# Supplementary material for: Characterization of Visceral and Subcutaneous Adipose Tissue Transcriptome and Biological Pathways in Pregnant and Non-Pregnant Women: Evidence for Pregnancy-Related Regional-Specific Differences in Adipose Tissue
Source: PLoS One. 2015 Dec 4;10(12):e0143779. doi: 10.1371/journal.pone.0143779 (PMC4670118; doi:10.1371/journal.pone.0143779)
Supplement: S3 Table — (DOC) [file pone.0143779.s010.doc]

**Table 4. A list of the 12 KEGG pathways that were significant in the comparison between visceral and subcutaneous adipose tissues of pregnant women**

| q-value | Odds Ratio | Genes in reference array, n | Genes in differentially expressed list, n | Map Name |
| --- | --- | --- | --- | --- |
| 0.000 | 5.55 | 72 | 16 | ECM-receptor interaction |
| 0.001 | 5.34 | 55 | 12 | PPAR signaling pathway |
| 0.001 | 4.89 | 64 | 13 | Protein digestion and absorption |
| 0.001 | 3.05 | 171 | 23 | Focal adhesion |
| 0.001 | 5.52 | 49 | 11 | Complement and coagulation cascades |
| 0.004 | 2.61 | 195 | 23 | Cytokine-cytokine receptor interaction |
| 0.007 | 4.02 | 63 | 11 | Arrhythmogenic right ventricular cardiomyopathy (ARVC) |
| 0.015 | 3.01 | 103 | 14 | Cell adhesion molecules (CAMs) |
| 0.015 | 4.71 | 40 | 8 | Malaria |
| 0.039 | 5.11 | 28 | 6 | Steroid hormone biosynthesis |
| 0.039 | 5.11 | 28 | 6 | African trypanosomiasis |
| 0.040 | 1.95 | 285 | 26 | Pathways in cancer |
