# Supplementary material for: Characterization of Visceral and Subcutaneous Adipose Tissue Transcriptome and Biological Pathways in Pregnant and Non-Pregnant Women: Evidence for Pregnancy-Related Regional-Specific Differences in Adipose Tissue
Source: PLoS One. 2015 Dec 4;10(12):e0143779. doi: 10.1371/journal.pone.0143779 (PMC4670118; doi:10.1371/journal.pone.0143779)
Supplement: S5 Table — (DOC) [file pone.0143779.s012.doc]

**Table 6. A list of the 26 enriched biological processes in the comparison between visceral and subcutaneous adipose tissues of non-pregnant women**

| **q-value** | **Odds Ration** | **Genes in reference array, n** | **Genes in differentially expressed list, n** | **Biological process** |
| --- | --- | --- | --- | --- |
| 0.000 | 26.80 | 23 | 7 | complement activation, classical pathway |
| 0.001 | 13.37 | 45 | 8 | protein activation cascade |
| 0.001 | 3.45 | 515 | 25 | cell adhesion |
| 0.002 | 33.68 | 14 | 5 | retinol metabolic process |
| 0.003 | 60.31 | 8 | 4 | retinal metabolic process |
| 0.004 | 2.02 | 3703 | 83 | single-multicellular organism process |
| 0.004 | 2.68 | 765 | 30 | localization of cell |
| 0.005 | 2.79 | 657 | 27 | circulatory system development |
| 0.009 | 17.82 | 22 | 5 | cellular response to cAMP |
| 0.010 | 2.02 | 2336 | 59 | developmental process |
| 0.011 | 7.11 | 77 | 8 | humoral immune response |
| 0.011 | 89.99 | 5 | 3 | acylglycerol acyl-chain remodeling |
| 0.014 | 2.86 | 489 | 21 | regulation of body fluid levels |
| 0.014 | 3.03 | 416 | 19 | tissue morphogenesis |
| 0.017 | 21.92 | 15 | 4 | regulation of complement activation |
| 0.017 | 6.28 | 86 | 8 | acute inflammatory response |
| 0.018 | 2.93 | 433 | 19 | vasculature development |
| 0.022 | 5.90 | 91 | 8 | response to purine-containing compound |
| 0.022 | 8.68 | 48 | 6 | organ formation |
| 0.029 | 6.57 | 72 | 7 | immunoglobulin mediated immune response |
