# Supplementary material for: Characterization of Visceral and Subcutaneous Adipose Tissue Transcriptome and Biological Pathways in Pregnant and Non-Pregnant Women: Evidence for Pregnancy-Related Regional-Specific Differences in Adipose Tissue
Source: PLoS One. 2015 Dec 4;10(12):e0143779. doi: 10.1371/journal.pone.0143779 (PMC4670118; doi:10.1371/journal.pone.0143779)
Supplement: S6 Table — (DOC) [file pone.0143779.s013.doc]

Table 7. A list of the 57 differentially expressed transcripts in the subcutaneous adipose tissues between the pregnant and non-pregnant women.

| **q-value** | **Fold Change*** | **Gene Name** | **SYMBOL** | **ENTREZ ID** |
| --- | --- | --- | --- | --- |
| 0.031 | 2.4 | complement component 6 | C6 | 729 |
| 0.049 | 2.2 | calcium binding protein 39-like | CAB39L | 81617 |
| 0.097 | -2.1 | apolipoprotein L domain containing 1 | APOLD1 | 81575 |
| 0.087 | -2.1 | olfactory receptor, family 52, subfamily N, member 5 | OR52N5 | 390075 |
| 0.094 | 2.0 | MOP-1 | MOP-1 | 643616 |
| 0.049 | 2.0 | V-set and immunoglobulin domain containing 4 | VSIG4 | 11326 |
| 0.069 | -1.9 | olfactory receptor, family 52, subfamily N, member 1 | OR52N1 | 79473 |
| 0.094 | 1.9 | membrane-spanning 4-domains, subfamily A, member 4 | MS4A4A | 51338 |
| 0.015 | -1.9 | hexokinase 2 | HK2 | 3099 |
| 0.031 | 1.8 | G protein-coupled receptor 64 | GPR64 | 10149 |
| 0.058 | -1.8 | FOS-like antigen 1 | FOSL1 | 8061 |
| 0.058 | -1.8 | solute carrier family 29 (nucleoside transporters), member 4 | SLC29A4 | 222962 |
| 0.049 | 1.8 | complement component 1, q subcomponent, A chain | C1QA | 712 |
| 0.097 | -1.8 | inositol 1,4,5-trisphosphate 3-kinase C | ITPKC | 80271 |
| 0.055 | -1.8 | serum/glucocorticoid regulated kinase 2 | SGK2 | 10110 |
| 0.058 | 1.8 | solute carrier family 40 (iron-regulated transporter), member 1 | SLC40A1 | 30061 |
| 0.067 | 1.8 | spondin 1, extracellular matrix protein | SPON1 | 10418 |
| 0.031 | -1.7 | cysteine dioxygenase, type I | CDO1 | 1036 |
| 0.029 | 1.7 | ATPase, Na+/K+ transporting, alpha 2 (+) polypeptide | ATP1A2 | 477 |
| 0.049 | 1.7 | complement component 1, q subcomponent, C chain | C1QC | 714 |
| 0.035 | -1.7 | solute carrier family 25, member 33 | SLC25A33 | 642740 |
| 0.087 | -1.7 | diacylglycerol O-acyltransferase homolog 2 (mouse) | DGAT2 | 84649 |
| 0.035 | 1.7 | chromosome 10 open reading frame 72 | C10orf72 | 196740 |
| 0.058 | 1.7 | mannose receptor, C type 1 | MRC1 | 414308 |
| 0.015 | -1.7 | solute carrier organic anion transporter family, member 4A1 | SLCO4A1 | 28231 |
| 0.035 | 1.7 | hematopoietic prostaglandin D synthase | HPGDS | 27306 |
| 0.062 | 1.7 | lymphatic vessel endothelial hyaluronan receptor 1 | LYVE1 | 10894 |
| 0.058 | 1.7 | LIM domain only 3 (rhombotin-like 2) | LMO3 | 55885 |
| 0.058 | 1.7 | ATP-binding cassette, sub-family A (ABC1), member 6 | ABCA6 | 23460 |
| 0.097 | 1.7 | lysophosphatidic acid receptor 6 | LPAR6 | 10161 |
| 0.069 | 1.7 | glycerol-3-phosphate dehydrogenase 1-like | GPD1L | 23171 |
| 0.093 | 1.7 | CD209 molecule | CD209 | 30835 |
| 0.081 | 1.7 | NA | NA | NA |
| 0.095 | -1.6 | retinol dehydrogenase 5 (11-cis/9-cis) | RDH5 | 5959 |
| 0.074 | 1.6 | coagulation factor XIII, A1 polypeptide | F13A1 | 2162 |
| 0.058 | 1.6 | STON1-GTF2A1L readthrough transcript | STON1-GTF2A1L | 11037 |
| 0.063 | 1.6 | folate receptor 2 (fetal) | FOLR2 | 2350 |
| 0.058 | -1.6 | transmembrane protein 182 | TMEM182 | 130827 |
| 0.079 | -1.6 | UDP-Gal:betaGlcNAc beta 1,4- galactosyltransferase, polypeptide 6 | B4GALT6 | 100287499 |
| 0.051 | 1.6 | vesicle-associated membrane protein 8 (endobrevin) | VAMP8 | 8673 |
| 0.060 | 1.6 | platelet derived growth factor D | PDGFD | 80310 |
| 0.079 | 1.6 | complement component 1, q subcomponent, B chain | C1QB | 713 |
| 0.035 | -1.6 | snail homolog 1 (Drosophila) | SNAI1 | 6615 |
| 0.062 | -1.6 | SRY (sex determining region Y)-box 17 | SOX17 | 64321 |
| 0.023 | 1.6 | histone cluster 1, H2bk | HIST1H2BK | 54145 |
| 0.049 | 1.6 | sushi-repeat-containing protein, X-linked | SRPX | 8406 |
| 0.079 | 1.5 | aldehyde oxidase 1 | AOX1 | 316 |
| 0.069 | 1.5 | glycine amidinotransferase (L-arginine:glycine amidinotransferase) | GATM | 2628 |
| 0.060 | 1.5 | family with sequence similarity 134, member B | FAM134B | 54463 |
| 0.050 | 1.5 | colony stimulating factor 1 receptor | CSF1R | 1436 |
| 0.085 | 1.5 | phospholipid transfer protein | PLTP | 5360 |
| 0.049 | -1.5 | sterile alpha motif domain containing 4A | SAMD4A | 23034 |
| 0.015 | 1.5 | matrix metallopeptidase 14 (membrane-inserted) | MMP14 | 4323 |
| 0.035 | 1.5 | ArfGAP with dual PH domains 2 | ADAP2 | 55803 |
| 0.039 | 1.5 | myoferlin | MYOF | 26509 |
| 0.052 | 1.5 | gasdermin B | GSDMB | 55876 |
| 0.089 | -1.5 | CD93 molecule | CD93 | 22918 |

(*) The fold change represents the number of times the average expression in one group is higher than the one in the other group. Positive values mean higher expression in subcutaneous tissues of pregnant compared to non-pregnant women, while negative values represent higher expression subcutaneous tissues of non-pregnant women compared to pregnant women. Genes are ranked by absolute fold change.
