# Supplementary material for: Characterization of Visceral and Subcutaneous Adipose Tissue Transcriptome and Biological Pathways in Pregnant and Non-Pregnant Women: Evidence for Pregnancy-Related Regional-Specific Differences in Adipose Tissue
Source: PLoS One. 2015 Dec 4;10(12):e0143779. doi: 10.1371/journal.pone.0143779 (PMC4670118; doi:10.1371/journal.pone.0143779)
Supplement: S7 Table — (DOC) [file pone.0143779.s014.doc]

**Table 9. A list of the alternative splicing events associated with the regional differences of the adipose tissue of pregnant women**

| **Symbol** | **Gene Name** | **Transcript ID** | **Exon ID** | **Probeset ID** | **Diff. mean FIRMA** | **q-value** |
| --- | --- | --- | --- | --- | --- | --- |
| ABLIM1 | actin binding LIM protein 1 | 3307939 | 619043 | 3308001 | 2.851 | 0.000 |
| ADRA2C | adrenergic, alpha-2C-, receptor | 2716328 | 249900 | 2716338 | -2.288 | 0.004 |
| CRB2 ^#^ | crumbs homolog 2 (Drosophila) | 3188478 | 544486 | 3188501 | -2.842 | 0.000 |
| DAPK1 ^#^ | death-associated protein kinase 1 | 3177880 | 538110 | 3177903 | 3.061 | 0.000 |
| DCLK1 ^#^ | doublecortin-like kinase 1 | 3509473 | 742860 | 3509602 | -3.042 | 0.000 |
| DES* | desmin | 2528476 | 131889 | 2528483 | -2.660 | 0.007 |
| DES* | desmin | 2528476 | 131895 | 2528491 | -2.291 | 0.003 |
| FAIM3 | Fas apoptotic inhibitory molecule 3 | 2452977 | 84252 | 2452981 | -2.944 | 0.000 |
| GATA6 ^#^ | GATA binding protein 6 | 3781245 | 908232 | 3781284 | -2.220 | 0.000 |
| GFPT2^#^ | glutamine-fructose-6-phosphate transaminase 2 | 2890660 | 359171 | 2890703 | 2.247 | 0.000 |
| HAND2 | heart and neural crest derivatives expressed 2 | 2794075 | 298919 | 2794102 | -2.449 | 0.000 |
| HAS1 ^#^ | hyaluronan synthase 1 | 3869215 | 960742 | 3869220 | -2.122 | 0.000 |
| HSPG2 | heparan sulfate proteoglycan 2 | 2400793 | 52523 | 2400797 | -2.081 | 0.075 |
| KLK5 | kallikrein-related peptidase 5 | 3868753 | 960481 | 3868759 | -4.606 | 0.000 |
| KNDC1 *^*^ | kinase non-catalytic C-lobe domain (KIND) containing 1 | 3272566 | 596819 | 3272650 | -2.459 | 0.000 |
| KNDC1 * | kinase non-catalytic C-lobe domain (KIND) containing 1 | 3272566 | 596822 | 3272655 | -3.467 | 0.000 |
| LAMB2 | laminin, beta 2 (laminin S) | 2674047 | 223487 | 2674099 | -2.046 | 0.001 |
| LPAR5 | lysophosphatidic acid receptor 5 | 3442137 | 701054 | 3442138 | -2.266 | 0.001 |
| MARCKSL1 | MARCKS-like 1 | 2404999 | 55081 | 2405001 | -2.411 | 0.000 |
| MGLL^#^ | monoglyceride lipase | 2694001 | 236116 | 2694073 | -2.738 | 0.002 |
| NKX3-1 | NK3 homeobox 1 | 3127978 | 506935 | 3127986 | -2.127 | 0.000 |
| NNMT | nicotinamide N-methyltransferase | 3349858 | 644508 | 3349861 | -2.313 | 0.010 |
| NPNT^#^ | nephronectin | 2738378 | 263882 | 2738380 | 2.012 | 0.000 |
| PAMR1* ^#^ | peptidase domain containing associated with muscle regeneration 1 | 3369442 | 656516 | 3369495 | 2.856 | 0.000 |
| PAMR1 *^#^ | peptidase domain containing associated with muscle regeneration 1 | 3369442 | 656516 | 3369494 | 2.730 | 0.000 |
| PAPLN | papilin, proteoglycan-like sulfated glycoprotein | 3543539 | 763903 | 3543593 | -2.017 | 0.000 |
| PLLP^#^ | plasma membrane proteolipid (plasmolipin) | 3693141 | 855189 | 3693152 | 2.303 | 0.000 |
| PMP22 | peripheral myelin protein 22 | 3746574 | 887424 | 3746605 | 5.244 | 0.000 |
| PODXL ^#^* | podocalyxin-like | 3073013 | 472183 | 3073017 | -2.120 | 0.000 |
| PODXL ^#^* | podocalyxin-like | 3073013 | 472213 | 3073060 | 2.099 | 0.000 |
| RCAN2^#^ | regulator of calcineurin 2 | 2955691 | 399076 | 2955724 | 2.626 | 0.000 |
| RDH10 ^#^ | retinol dehydrogenase 10 (all-trans) | 3103293 | 491273 | 3103294 | 2.461 | 0.002 |
| SEMA3G ^#^ | sema domain, immunoglobulin domain (Ig), short basic domain, secreted, (semaphorin) 3G | 2676141 | 224606 | 2676157 | -2.259 | 0.000 |
| SLC38A1^#^ | solute carrier family 38, member 1 | 3452231 | 707206 | 3452302 | 2.273 | 0.000 |
| SLC7A8 ^#^* | solute carrier family 7 (cationic amino acid transporter, y+ system), member 8 | 3557209 | 772193 | 3557250 | -2.069 | 0.000 |
| SLC7A8 ^#^* | solute carrier family 7 (cationic amino acid transporter, y+ system), member 8 | 3557209 | 772200 | 3557261 | -2.042 | 0.000 |
| SPTBN1 | spectrin, beta, non-erythrocytic 1 | 2482505 | 103031 | 2482530 | -5.087 | 0.000 |
| SRRM2* | serine/arginine repetitive matrix 2 | 3645253 | 826444 | 3645274 | -2.564 | 0.001 |
| SRRM2* | serine/arginine repetitive matrix 2 | 3645253 | 826444 | 3645273 | -2.518 | 0.000 |
| TM4SF1^#^ | transmembrane 4 L six family member 1 | 2700365 | 240134 | 2700369 | -2.372 | 0.006 |
| TRNP1 | TMF1-regulated nuclear protein 1 | 2326846 | 7232 | 2326860 | -3.112 | 0.000 |
| TSPAN15 ^#^ | tetraspanin 15 | 3250373 | 582734 | 3250374 | -2.073 | 0.000 |

*Genes showing differential exon usage for two exons

# Genes demonstrating differential exon usage that were also differentially expressed
